# Sp1 is a substrate of Keap1 and regulates the activity of CRL4A^WDR23^ ubiquitin ligase toward Nrf2

**DOI:** 10.1016/j.jbc.2021.100704

**Published:** 2021-04-23

**Authors:** Ferbian Milas Siswanto, Ami Oguro, Susumu Imaoka

**Affiliations:** 1 Department of Biomedical Chemistry, School of Science and Technology, Kwansei Gakuin University, Sanda, Japan; 2 Program of Biomedical Science, Graduate School of Integrated Sciences for Life, Hiroshima University, Hiroshima, Japan

**Keywords:** specificity protein 1, nuclear factor 2 (erythroid-derived 2-like factor), WD40 repeat protein 23, Kelch-like ECH-associated protein 1, DNA damage-binding protein 1, Cullin4A, Ring-box 1, Cullin 4A-RING ligase, oxidative stress, posttranslational regulation, gene regulation, ARE, antioxidant response elements, CRL4, Cullin 4-RING ligase, CUL4A, Cullin 4A, DDB1, DNA damage-binding protein 1, Keap1, Kelch-like ECH-associated protein 1, KD, knockdown, HO-1, heme oxygenase-1, NQO1, NAD(P)H quinone dehydrogenase 1, Nrf2, nuclear factor erythroid 2-related factor 2, RBX1, Ring-box 1, shRNA, short hairpin RNA, Sp1, specificity protein 1, WDR23, WD40 repeat protein 23

## Abstract

Nuclear factor erythroid 2–related factor 2 (Nrf2) is a critical transcription factor that orchestrates cellular responses to oxidative stress. Because the dysregulation of Nrf2 has been implicated in many diseases, precise regulation of its protein level is crucial for maintaining homeostasis. Kelch-like-ECH-associated protein 1 (Keap1) and WD40 repeat protein 23 (WDR23) directly regulate Nrf2 levels *via* similar but distinct proteasome-dependent pathways. WDR23 forms a part of the WDR23-Cullin 4A-RING ubiquitin ligase complex (CRL4A^WDR23^), whereas Keap1 serves as a substrate adaptor for the Cullin 3–containing ubiquitin ligase complex. However, the mechanisms underlying crosstalk between these Keap1 and WDR23 pathways for the regulation of Nrf2 levels have not been investigated. Here, we showed that knockdown (KD) of Keap1 upregulated the expression of Cullin4A (*CUL4A*) in a specificity protein 1 (Sp1)–dependent manner. We also revealed that Sp1 interacted with Keap1, leading to ubiquitination of Sp1. Increases in Sp1 by Keap1 KD triggered Sp1 binding to the fourth Sp1 binding site (Sp1_M4) within the −230/+50 region of the *CUL4A* gene. We also demonstrated that the overexpression and KD of Sp1 reduced and increased Nrf2 protein levels, respectively. These effects were abrogated by the WDR23 KD, suggesting that Sp1 also regulates Nrf2 levels *via* the ubiquitin ligase complex CRL4A^WDR23^. In conclusion, we discovered Sp1 as a novel substrate of Keap1 and provided evidence that Sp1 regulates the expression of *CUL4A*. We revealed a novel role for Sp1 in mediating crosstalk between two independent regulators of Nrf2 protein levels.

The transcription factor nuclear factor erythroid 2–related factor 2 (Nrf2) is a central regulator of cellular redox homeostasis. It is beneficial for cytoprotection against harmful extrinsic and intrinsic insults, such as xenobiotics and oxidative stress. Under basal conditions, Nrf2 mRNA is constitutively expressed (1), and the abundance of Nrf2 protein levels is regulated at the protein level by Kelch-like ECH-associated protein 1 (Keap1), a substrate adaptor protein for the Cullin 3-containing E3 ubiquitin ligase complex (2). Keap1 is a cysteine-rich protein, and human Keap1 has 27 cysteine residues that function as sensors of various oxidants and electrophiles (3). Homodimeric Keap1 binding to monomeric Nrf2 through the ETGE and DLG motifs promotes Nrf2 degradation by the ubiquitin-proteasome pathway, thereby maintaining low cellular levels of Nrf2. The cysteine residues in Keap1 are modified by oxidative stress, which results in conformational changes in Keap1 and releases the weaker interaction with the DLG motif (4, 5). Although Keap1–ETGE binding is preserved, the ubiquitination of Nrf2 does not occur, providing time for newly synthesized Nrf2 to accumulate and translocate into the nucleus. In the nucleus, Nrf2 heterodimerizes with small Maf protein family members and binds to antioxidant response elements (AREs) or electrophile responsive elements located in the regulatory regions of antioxidative and phase II cytoprotective genes, such as heme oxygenase-1 (HO-1) and NAD(P)H quinone dehydrogenase 1 (NQO1) (6, 7).

In addition to the canonical Keap1 pathway, β-transducin repeat-containing protein and HMG-CoA reductase degradation 1 homolog have been reported to induce proteasome-dependent Nrf2 degradation in a Keap1-independent manner (8, 9). WD40 repeat protein 23 (WDR23) was recently shown to function as a regulator of Nrf2 levels and activity independently of Keap1 (10, 11). Additionally, we found that WDR23 regulates the expression of Nrf2-driven drug-metabolizing enzymes (12). WDR23, also known as DNA damage-binding protein 1 (DDB1) and Cullin4-associated factor 11 in mammalian cells, is a substrate recognition protein for the Cullin 4-RING ligase (CRL4) complex that consists of CUL4 as a scaffold protein, DDB1 as an adaptor protein, and Ring-box 1 (RBX1) as a RING-finger protein (13). Mammalian WDR23 possesses two isoforms produced by alternative splicing, isoforms 1 and 2, with isoform 2 being deficient in the 45 to 70th amino acids of isoform 1. WDR23 isoform 1, which primarily localizes in the cytoplasm, regulates cytoplasmic proteins, whereas isoform 2 predominantly resides in the nucleus to inhibit the activity of nuclear factors by promoting protein turnover (14). WDR23 targets proteins for degradation *via* ubiquitination, including Holliday junction resolvase GEN-1 (14), p21 (15), stem-loop binding protein (16), and Nrf2 (11).

The transient activation of Nrf2 in normal cells is beneficial for cytoprotection and the prevention of pathological conditions; however, its consecutive activation in cancer cells is responsible for chemoresistance and is associated with a poor prognosis (17). Therefore, the precise regulation of Nrf2 levels is crucial. A somatic mutation in highly conserved Kelch or the intervening region domain of the Keap1 protein that results in the constitutive activation of Nrf2 often occurs in cancer cells (18, 19). Therefore, the second layer of Nrf2 regulation is important for preventing carcinogenesis and chemoresistance. We previously reported that the knockdown (KD) of WDR23 was sufficient to increase the level and transactivity of Nrf2, whereas its overexpression only affected Nrf2 under Keap1 KD (12). These findings indicate that WDR23 regulates Nrf2 under basal conditions, whereas the further induction of WDR23 activity toward Nrf2 requires the inhibition of Keap1. Therefore, WDR23 plays a major role in the regulation of Nrf2 in cancer cells bearing *Keap1* mutation. However, the molecular mechanisms underlying crosstalk between these two independent and parallel regulators of Nrf2, particularly that by which WDR23 senses the function of Keap1, have not yet been elucidated.

Specificity protein 1 (Sp1) is a ubiquitously expressed nuclear transcription factor belonging to the C2H2-type zinc-finger protein family. Sp1 regulates gene expression *via* protein–protein interactions, such as with vascular endothelial growth factor receptor-2 (20), or acts in concert with other transcription factors, including Stat1 (21), nuclear factor-κB (22), and EGR-1 (23), in the absence of the TATA box. It binds to the sequence known as the GC box (GGGCGG or CCGCCC) in the promoters of numerous genes with high affinity (24, 25). Sp1 was initially regarded as a coordinator of the constitutive expression of housekeeping genes; however, recent studies showed that Sp1 responded to physiological and pathological stimuli (26, 27). Previous findings clearly demonstrated that Sp1 protein levels and transcriptional activity were induced by oxidative stress (28, 29, 30). For example, we found that high glucose-induced oxidative stress increased nuclear Sp1 levels, which inhibited the expression of *sEH* (27). Increases in the level and activity of Sp1 have been widely proven to be responsible for oxidative stress-related carcinogenesis, including proliferation, the cell cycle, invasion, metastasis, angiogenesis, and the inhibition of apoptosis in hepatocellular carcinoma (31). Although Sp1 plays a role in the oxidative stress response pathway, the underlying molecular mechanisms have not yet been elucidated in detail.

We herein demonstrated the role of Sp1 as a mediator of Keap1–WDR23 crosstalk for the regulation of Nrf2. The results obtained herein revealed that Keap1 directly regulates Sp1. The stabilization of Sp1 during the KD of Keap1 resulted in the transcriptional activation of Cullin4A (*CUL4A*) and induced the WDR23-dependent regulation of Nrf2.

## Results

### Effects of the KD of Keap1 on the expression of CRL4A^WDR23^ components

**Figure 1:**
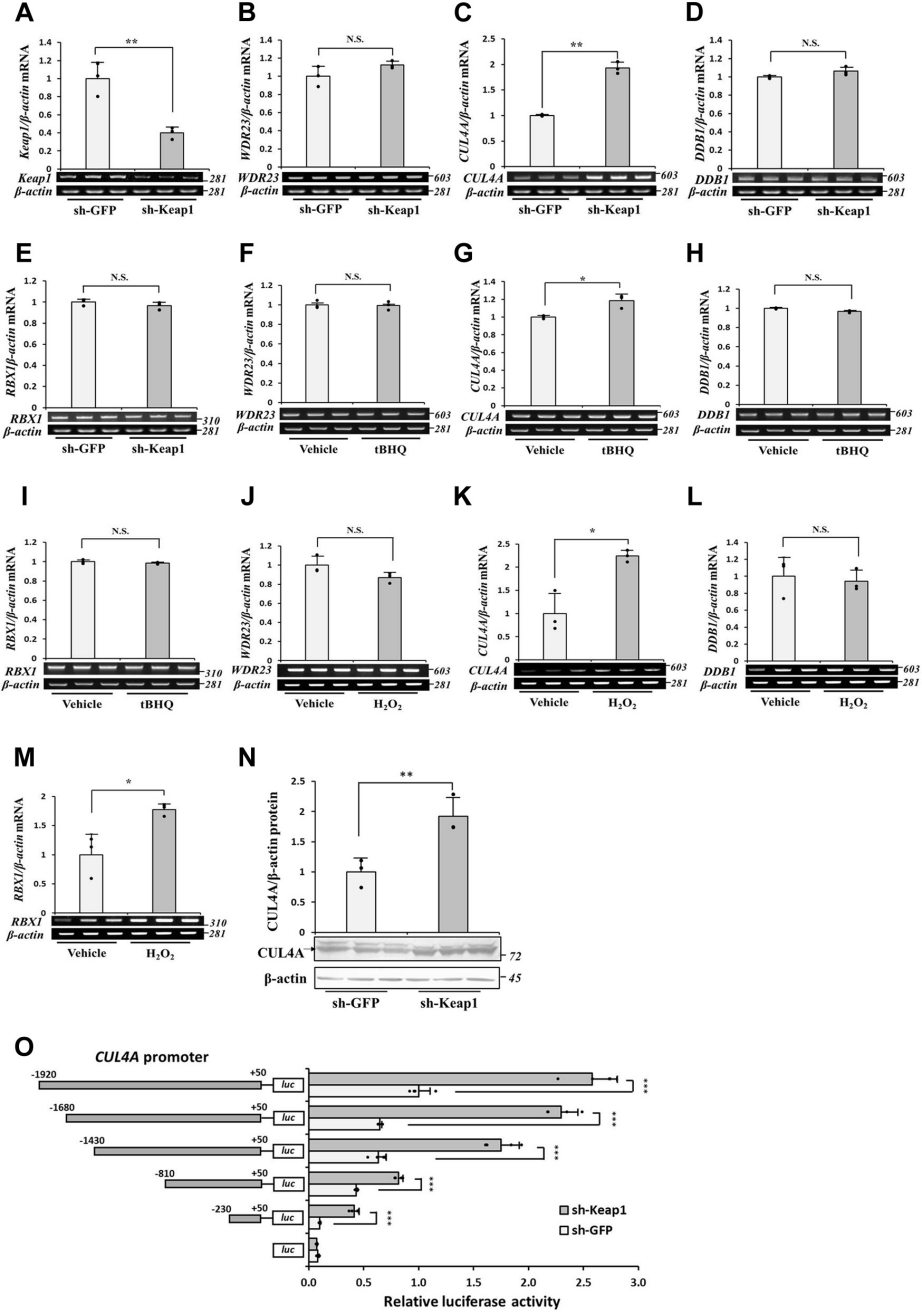
**The knockdown of Keap1 increases transcript and protein levels of CUL4A.***A–E*, Keap1 knockdown cells were generated using shRNA in Hep3B cells, and the mRNA expression levels of *Keap1*, *WDR23*, *CUL4A*, *DDB1*, and *RBX1* were measured by RT-PCR. *F*–*I*, Hep3B cells were grown in the presence or absence of tBHQ (60 μM; 8 h), and the abundance of mRNA was examined by RT-PCR. *J*–*M*, Hep3B cells were treated with H_2_O_2_ (100 μM; 8 h), and mRNA expression levels were assessed by RT-PCR. *N*, total cell lysates from control (sh-GFP) and Keap1 KD (sh-Keap1) cells were subjected to immunoblotting against the anti-CUL4 antibody. *O*, the upstream region −1920/+50 of *CUL4A* in pGL3 or its 5′-endo deletion construct was co-transfected with pRL-null as an internal control plasmid into control and Keap1 KD Hep3B cells. Luciferase activity was assessed 48 h after transfection using the Dual-Luciferase Reporter Assay system relative to the promoter-less construct pGL3-Basic. Results are shown as a ratio relative to the activity of *CUL4A* promoter −1920/+50 in control cells. All values are means ± SD from three independent experiments. N.S. not significant, ∗*p* < 0.05, ∗∗*p* < 0.01, ∗∗∗*p* < 0.001 *versus* the indicated cells. CUL4A, Cullin4A; DDB1, DNA damage-binding protein 1; KD, knockdown; Keap1, Kelch-like ECH-associated protein 1; RBX1, Ring-box 1; shRNA, short hairpin RNA; tBHQ, tert-butylhydroquinone; WDR23, WD40 repeat protein 23.

The identification of WDR23 as a novel regulator of Nrf2 in addition to the well-established Keap1 pathway has attracted interest because of its potential to enhance the stress response, refine the type of stress response induced, or control tissue specificity (11). However, the mechanisms by which Keap1 and WDR23 signaling interact with each other remain unclear. Since we previously reported that WDR23 activity was limited to Keap1 (12), we initially examined the effects of the KD of Keap1 on the expression of CRL4A^WDR23^ components, including *WDR23*, *CUL4A*, *DDB1*, and *RBX1*. The short hairpin RNA (shRNA)-mediated KD of Keap1 significantly decreased (∼60%) Keap1 mRNA levels, suggesting successful KD (Fig. 1*A*). The present results showed that the KD of Keap1 upregulated the expression of *CUL4A* but not *WDR23*, *DDB1*, or *RBX1* (Fig. 1, *B*–*E*). Similarly, the chemical inhibition of Keap1 by tert-butylhydroquinone (tBHQ) increased *CUL4A* mRNA levels, whereas those of *WDR23*, *DDB1*, and *RBX1* remained unchanged (Fig. 1, *F*–*I*). Because Keap1 is an intracellular sensor of oxidative and electrophilic insults, we hypothesized that the effects of the KD of Keap1 on the expression of CRL4A^WDR23^ components may be mimicked by hydrogen peroxide, which is an oxidizing agent that disrupts the Keap1–Nrf2 interaction. We found that the expression of *CUL4A* was also increased by the H_2_O_2_ treatment (Fig. 1, *J*–*M*). We then examined the effects of the KD of Keap1 on the protein levels of CUL4A and found that they were elevated (Fig. 1*N*).

To elucidate the mechanisms underlying the regulation of *CUL4A* expression by Keap1, we investigated *CUL4A* promoter activity under Keap1 KD conditions using a luciferase reporter assay. Luciferase reporter plasmids (pGL3-basic) containing −1920/+50 bp of the *CUL4A* genomic region were constructed and transfected into Hep3B cells. The KD of Keap1 induced a 2.5-fold increase in promoter activity (Fig. 1*O*). To identify the promoter region required for the transcriptional activation of *CUL4A*, a deletion analysis of the promoter was performed. Basal promoter activity was gradually decreased by the consecutive 5′-end deletion of the upstream region of *CUL4A*. However, the KD of Keap1 still enhanced the promoter activity of the −230/+50 construct, suggesting that the regulatory region of *CUL4A* by Keap1 is present within this segment.

### The role of Sp1 on the Keap1 KD-mediated induction of CUL4A

**Figure 2:**
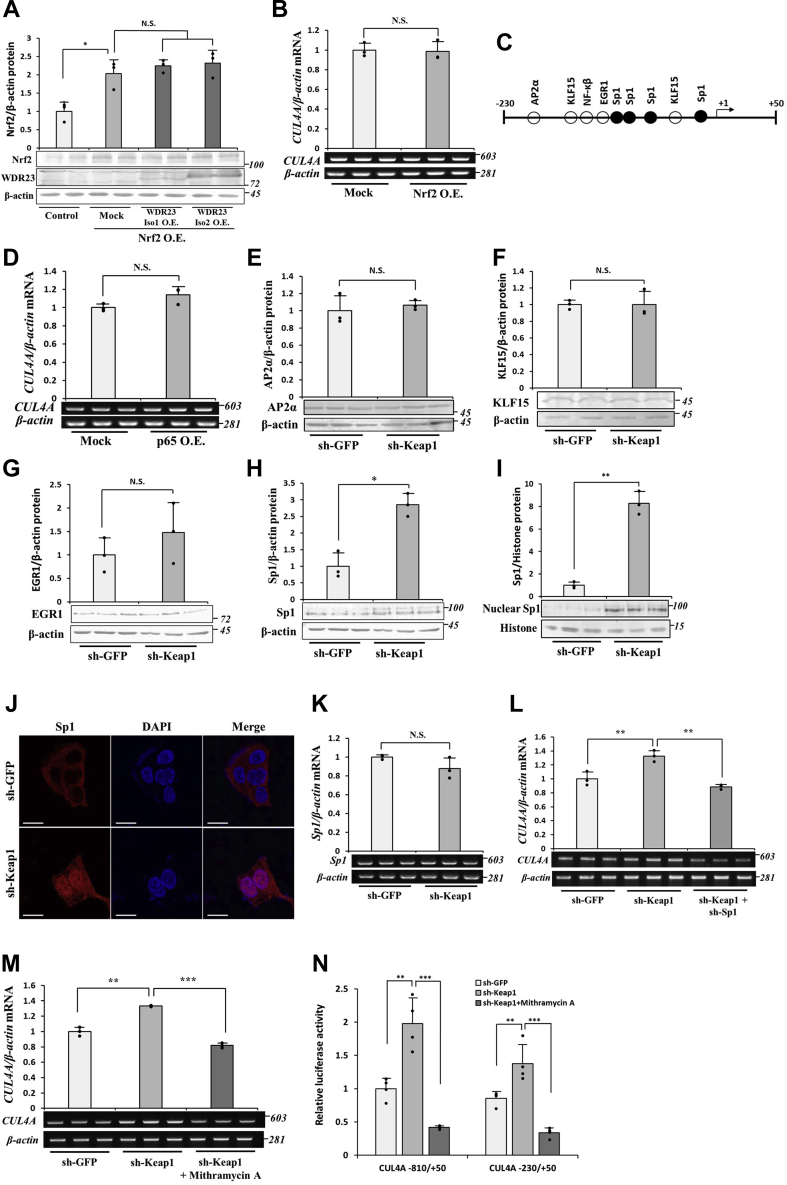
**The requirement of Sp1 in Keap1 KD-induced *CUL4A* expression.***A*, Nrf2 and WDR23 protein levels were assessed by immunoblotting using the cell lysates from control (pcDNA-Mock only), mock (pcDNA-Mock and pcDNA-Nrf2), and WDR23 OE (pcDNA-WDR23 isoform 1 or 2 and pcDNA-Nrf2) cells. *B*, the abundance of mRNA encoding CUL4A was assessed from Mock and Nrf2-overexpressing cells by RT-PCR. *C*, schematic representation of the −230/+50 of human *CUL4A*, with +1 representing the transcription start site. The position of transcription factor-binding sites was predicted by the JASPAR database. *D*, p65 in pcDNA3.1(−) was transfected in Hep3B cells, and the mRNA level of *CUL4A* was evaluated by RT-PCR. *E*–*H*, the abundance of the AP2α, KLF15, EGR1, and Sp1 proteins was examined in whole cell lysates from control (sh-GFP) and Keap1 KD (sh-Keap1) Hep3B cells by immunoblotting. *I*, the abundance of the nuclear Sp1 from control and Keap1 KD Hep3B cells was examined by immunoblotting. *J*, cellular localization of Sp1 (*red*) was examined by immunofluorescence counterstained with DAPI staining (*blue*) to indicate the location of the nucleus in the control and Keap1 KD Hep3B cells, scale bar: 20 μm. *K*, Sp1 mRNA abundance was measured by RT-PCR. *L* and *M*, the effects of Sp1 KD or mithramycin A (100 nM, 6 h) on the *CUL4A* mRNA levels of Keap1 KD cells were investigated by RT-PCR. *N*, control and Keap1 KD Hep3B cells in the presence or absence of mithramycin A (100 nM, 6 h) were transfected with pGL3-containing *CUL4A* promoter −810/+50 or −230/+50. Luciferase activity was measured 48-h posttransfection. Graphs are means ± SD from three independent experiments. N.S. not significant, ∗*p* < 0.05, ∗∗*p* < 0.01, ∗∗∗*p* < 0.001 *versus* the indicated cells. CUL4A, Cullin4A; KD, knockdown; OE, overexpression; Keap1, Kelch-like ECH-associated protein 1; Nrf2, Nuclear factor erythroid 2-related factor 2; Sp1, specificity protein 1; WDR23, WD40 Repeat protein 23.

To elucidate the transcriptional regulation of *CUL4A*, particularly with the KD of Keap1, we attempted to identify the relevant transcription factor(s) responsible for the regulation of *CUL4A* transcription. Nrf2 protein levels were significantly increased by the KD of Keap1 because ofdue to the depletion of Nrf2 ubiquitination, indicating that the WDR23 pathway might be activated by Nrf2 as a self-regulatory feedback mechanism. However, we found that the overexpression of WDR23 did not affect Nrf2 protein levels in Nrf2-overexpressing cells with intact Keap1 function (Fig. 2*A*). Furthermore, the overexpression of Nrf2 alone did not change the mRNA expression of any component of *CUL4A* (Fig. 2*B*). These results support the idea that activity of the WDR23 pathway being limited to the function of Keap1.

We then predicted several putative transcription factor–binding sites using the JASPAR database. We found the putative binding sites of AP2α, KLF15, EGR1, NF-κβ, and Sp1 within −230/+50 bp of *CUL4A* (Fig. 2*C*). Among all possible transcription factors, only NF-κβ has been reported as a downstream target of Keap1. Because Keap1 directly interacts with and induces the autophagic degradation of IKKβ, its depletion results in the upregulation of nuclear factor-κB (32, 33). However, the overexpression of p65 did not change the expression of *CUL4A* (Fig. 2*D*), suggesting that the effects of the KD of Keap1 on *CUL4A* were independent of NF-κβ. We then observed the protein levels of other potential transcription factors under the KD of Keap1. AP2α, KLF15, and EGR1 protein levels were unaffected by the KD of Keap1 (Fig. 2, *E*–*G*, respectively). In contrast, total and nuclear Sp1 protein levels were elevated (Fig. 2, *H* and *I*). In Keap1 KD cells, Sp1 accumulated in the nucleus (Fig. 2*J*). The mRNA levels of Sp1 were unaltered by the KD of Keap1 (Fig. 2*K*), suggesting that the Sp1 elevation induced by the KD of Keap1 occurred at the posttranscriptional level.

To confirm the involvement of Sp1 on Keap1 KD-induced *CUL4A* expression, we inhibited the activity of Sp1 using a genetic and chemical approach. The shRNA-mediated genetic inhibition of Sp1 in Keap1 KD Hep3B cells attenuated the upregulated expression of *CUL4A* (Fig. 2*L*). Similarly, the treatment of Keap1 KD cells with mithramycin A (100 nM, 6 h), a potent chemical inhibitor of Sp1, reduced the expression of *CUL4A* (Fig. 2*M*). This is consistent with the results showing that mithramycin A abolished Keap1 KD–induced increases in *CUL4A* promoter activity (Fig. 2*N*). Collectively, these results support the role of Sp1 in the regulation of *CUL4A* expression under Keap1 KD conditions.

### Transcriptional regulation of the CUL4A promoter by Sp1

**Figure 3:**
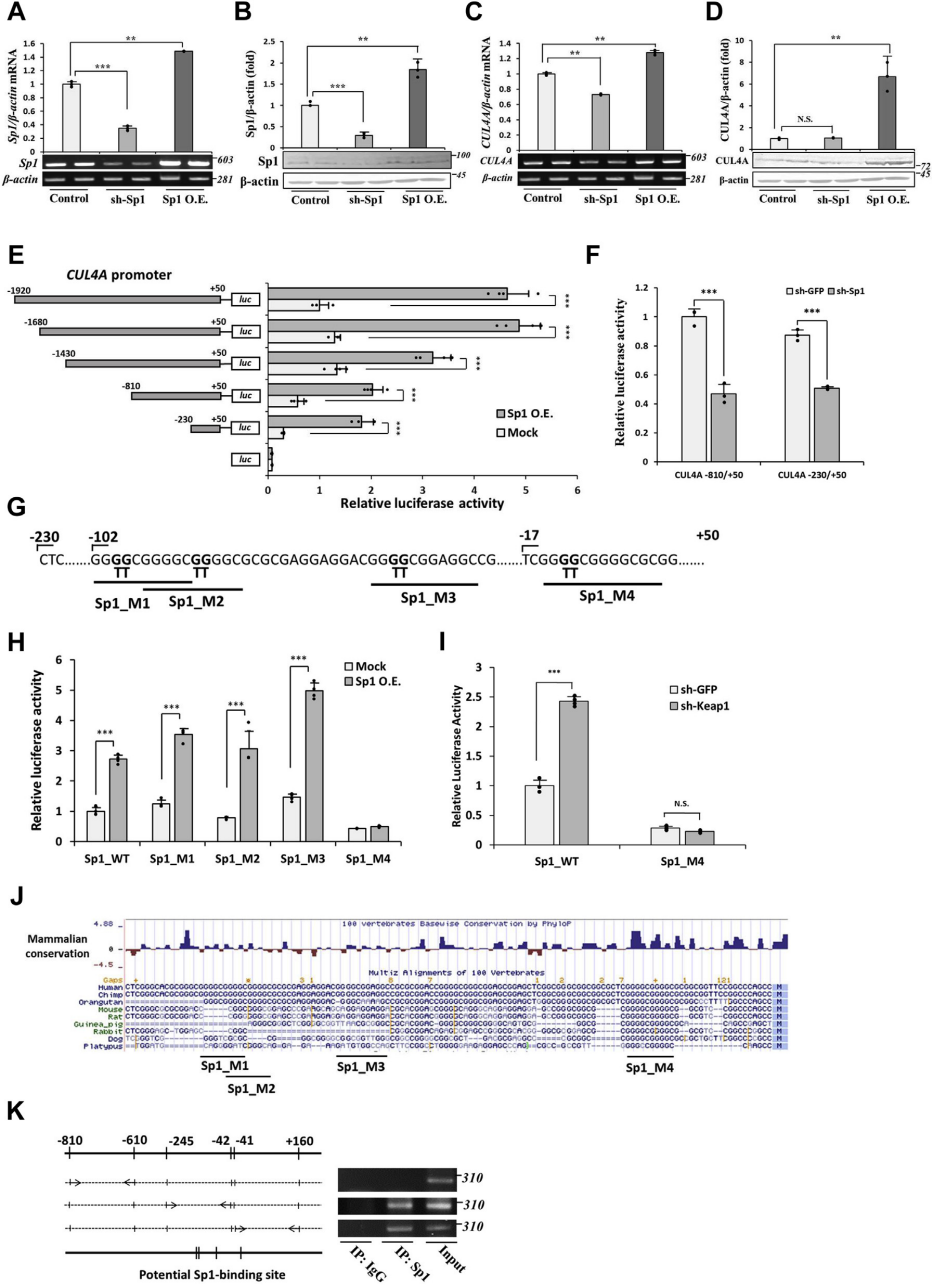
**Sp1 regulates *CUL4A* expression and promoter activity.***A* and *B*, Sp1 KD cells were generated using shRNA in Hep3B cells, and overexpression was achieved by the transfection of Sp1 in pCMV-Myc. The abundance of mRNA and the protein levels of Sp1 from control, Sp1 KD, and Sp1 OE cells were evaluated by RT-PCR and immunoblotting, respectively, to confirm the efficiency of KD and OE. *C* and *D*, the mRNA and protein levels of CUL4A from Sp1 KD and overexpressing cells were examined by RT-PCR and immunoblotting, respectively. *E*, the upstream region −1920/+50 of *CUL4A* in pGL3 or its consecutive 5′-end deletion construct was co-transfected with pRL-null and pCMV-Mock or pCMV-Sp1 in Hep3B cells. Luciferase activity was assessed 48 h posttransfection. *F*, control (sh-GFP) and Sp1 KD (sh-Sp1) Hep3B cells were transfected with pGL3-containing *CUL4A* promoter −810/+50 or −230/+50. Luciferase activity was assessed 48-h posttransfection. *G*, Sp1-binding sites and its mutated construct (Sp1_M1–Sp1_M4) within the *CUL4A* promoter −230/+50. *H*, WT *CUL4A* promoter −230/+50 or the mutant construct, in which each Sp1-binding motif was mutated, was co-transfected with pRL-null and pCMV-Mock or pCMV-Sp1 in Hep3B cells, and luciferase activity was measured. *I*, WT *CUL4A* promoter −230/+50 or the Sp1_M4 mutant construct was co-transfected with pRL-null in control (sh-GFP) or Keap KD (sh-Keap1) Hep3B cells, and luciferase activity was measured. Graphs are means ± SD from three independent experiments. ∗*p* < 0.05, ∗∗*p* < 0.01, ∗∗∗*p* < 0.001 *versus* the indicated cells. *J*, the sequences of the *CUL4A* promoter from 100 vertebrate species were mapped onto the human *CUL4A* promoter built on the UCSC genome browser. Phylogenetic *p*-values indicate conservation. *K*, *left panel*, Genomic positions of regions that were selected for a PCR-based analysis in the ChIP assay. The indicated Sp1-binding sites within the *CUL4A* promoter were predicted by the JASPAR database. *Right panel*, ChIP assay with an Sp1 antibody or control mouse IgG, with input chromatin as the positive control. After reverse crosslinking, DNA was amplified using the indicated primer sets. CUL4A, Cullin4A; KD, knockdown; OE, overexpression; Keap1, Kelch-like ECH-associated protein 1; Sp1, specificity protein 1.

Sp1 is a ubiquitously expressed transcription factor that regulates the expression of numerous genes. To further investigate the role of Sp1 on *CUL4A* gene expression, we analyzed the expression of CUL4A at the transcript and protein levels after the KD or ectopic overexpression of Sp1 (Fig. 3, *A* and *B*). We found that the KD of Sp1 significantly decreased *CUL4A* mRNA levels, which slightly reduced CUL4A protein levels. In contrast, the overexpression of Sp1 elevated *CUL4A* mRNA levels and induced an approximately 7-fold increase in the accumulation of the CUL4A protein (Fig. 3, *C* and *D*), suggesting that Sp1 contributes to the expression of *CUL4A*. These results indicate that Sp1 regulates the basal and inducible expression of *CUL4A*. We then performed a luciferase reporter assay to further confirm the results outlined above. Similar to the phenomena observed under Keap1 KD conditions, the overexpression of Sp1 significantly enhanced the luciferase activity of the pGL3-containing −1920/+50 bp of the *CUL4A* genomic region. Constructs containing a series of 5′ end deletions were all responsive to the overexpression of Sp1 (Fig. 3*E*). In contrast, the KD of Sp1 markedly decreased the promoter activity of pGL3-containing −810/+50 bp and −230/+50 bp of the *CUL4A* genomic region construct by approximately 50% (Fig. 3*F*).

Within the *CUL4A* promoter −230/+50 region, four Sp1-binding sites were identified using the JASPAR database. To verify the contribution of Sp1-binding sites to the functional activity of the proximal *CUL4A* promoter, each Sp1 putative site within the −230/+50 construct was individually mutated (Fig. 3*G*). Because Sp1 is known to bind to various consensus sequences, we simply replaced the core GG sequences with TT. The mutant constructs Sp1_M1, Sp1_M2, and Sp1_M3 exhibited similar basal promoter activities to the WT construct, whereas Sp1_M4 basal promoter activity was weaker than that of the WT. The mutation in the fourth site (Sp1_M4) suppressed the activation of promoter activity by the overexpression of Sp1, suggesting its crucial role for the Sp1-activated *CUL4A* promoter (Fig. 3*H*). The mutation in the fourth site also abrogated the response of the *CUL4A* promoter to the KD of Keap1 (Fig. 3*I*), which is consistent with the results described above, suggesting that Sp1 binding to the fourth putative site plays an important role in Keap1 KD–induced *CUL4A* expression. We consistently found that the fourth Sp1-binding site was highly conserved in mammals, and based on multiple species alignment phylogenetic *p*-values, the conservation of the fourth site was significantly greater than that of the other sites (Fig. 3*J*).

Based on the result showing that Sp1 plays an important role in the regulation of *CUL4A* promoter activity, we were prompted to investigate whether this transcription factor regulates the expression of *CUL4A* at the transcriptional level by directly binding to its promoter. The ChIP assay was conducted using the Sp1 antibody in Hep3B cells and detected *CUL4A* promoter-bound DNA fragments using specific primers. ChIP PCR products were identified using primers that amplified regions −230 to −42 and −41 to +160 of the *CUL4A* gene, but not with primers that amplified regions −810 to −610 with no potential Sp1-binding sites (Fig. 3*K*). The input was unfragmented chromatin as a positive control in PCR for each segment of interest.

### Direct interaction of Keap1 with Sp1

**Figure 4:**
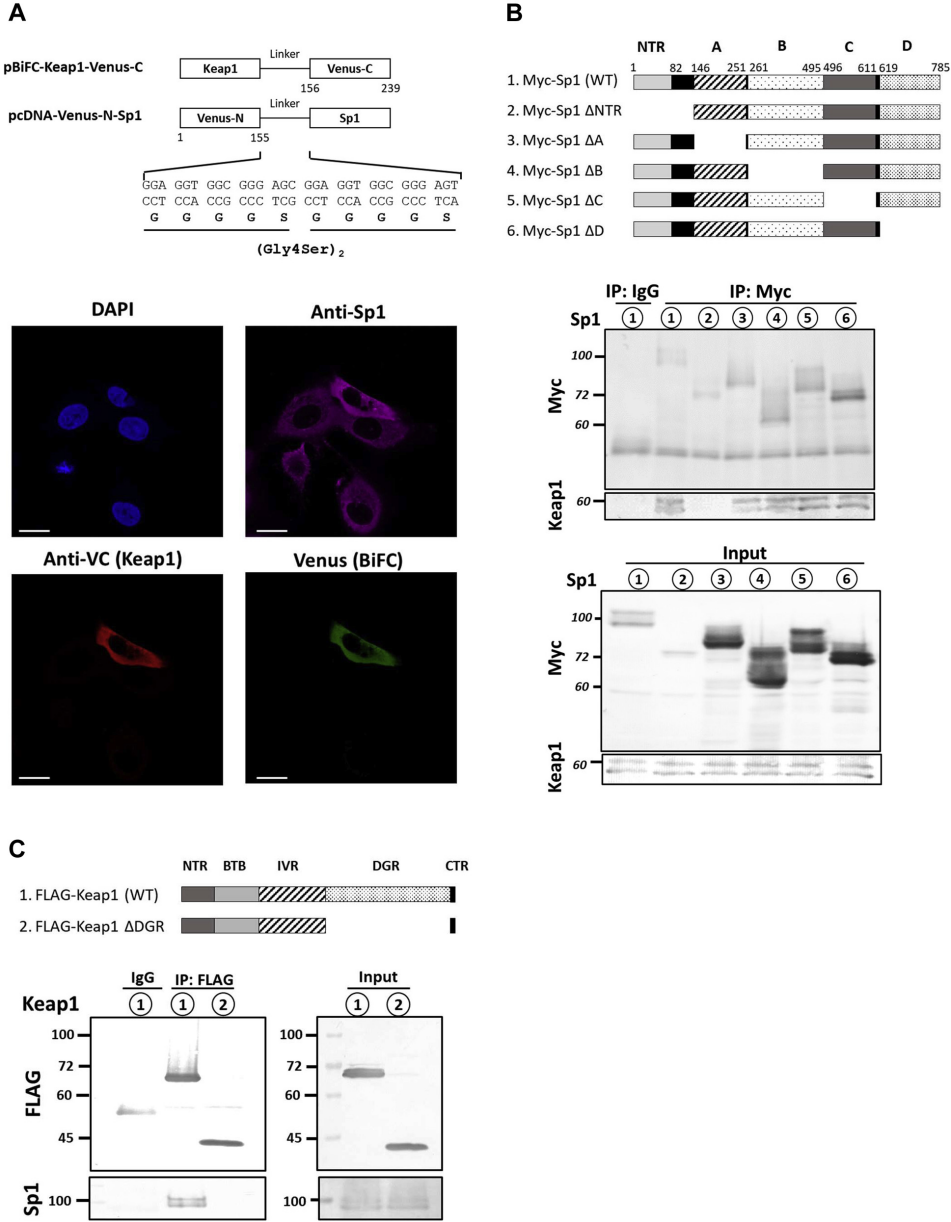
**Direct interaction of Keap1 with Sp1.***A*, N- and C-terminal fragments of Venus fluorescent proteins were fused to the N terminal of Sp1 and C terminal of Keap1, respectively (*upper panel*). The interaction between Sp1 and Keap1 brings the N- and C-terminal fragments in proximity to reconstitute an intact fluorescent protein. VN-Sp1 was co-expressed with Keap1-VC in Hep3B cells, and overexpression was confirmed with an anti-Sp1 or anti-VC antibody. Venus fluorescence as the BiFC signal indicates the interaction (*below panel*). Scale bar: 20 μm. *B*, the immunoprecipitation assay was performed to evaluate the domain responsible for the binding of Sp1 with Keap1. Hep3B cells were transfected with Myc-Sp1 full-length or domain-truncated mutants as indicated in the diagram (*upper panel*). Transfected cells were immunoprecipitated with an anti-Myc antibody, and immunoprecipitated proteins were subjected to an immunoblotting analysis with anti-Myc and anti-Keap1 antibodies. NTR, N-terminal region; A, Transactivation domain A; B, Transactivation domain B; C, Transactivation domain C; D, domain D. *C*, Hep3B cells were transfected with the FLAG-Keap1 full-length or ΔDGR mutant as indicated in the diagram (*upper panel*). Transfected cells were immunoprecipitated with an anti-FLAG antibody, and immunoprecipitated proteins were subjected to an immunoblotting analysis with an anti-FLAG and anti-Sp1 antibody. BiFC, bimolecular fluorescence complementation; BTB, Bric-a-Brac; CTR, C-terminal region; DGR, double glycine repeat; Keap1, Kelch-like ECH-associated protein 1; IVR, intervening region; NTR, N-terminal region; Sp1, specificity protein 1.

Because we found that Sp1 protein levels were increased by the KD of Keap1 and that Sp1 mediates the activation of Keap1 KD-induced *CUL4A* expression, we postulated that Sp1 is directly bound to and regulated by Keap1. To test this hypothesis, we investigated the intracellular interaction of Sp1 with Keap1 using a bimolecular fluorescence complementation (BiFC) assay. BiFC relies on the principle that interacting proteins tagged with the nonfluorescent N- or C-terminal fragments of a β-barrel fluorescent protein enable the fragments of the fluorescent protein to fuse and refold, leading to the acquisition of a fluorescent complex (34). When the N-terminal fragment of Venus (VN155, I152 L) fused to the N terminal of Sp1 was co-expressed with Keap1 fused to the C-terminal fragment of Venus (VC155) in Hep3B cells, Venus fluorescent was mainly detected in the cytosol as a BiFC signal, suggesting the direct interaction of Sp1 with Keap1 (Fig. 4*A*). To clarify which domain of Sp1 and Keap1 contribute to this interaction, we investigated the interaction of full-length and several truncated Sp1 and Keap1 mutants using an immunoprecipitation assay. Keap1 was present in the lysate pulled down with the Myc antibody in full-length Sp1-overexpressing cells. Keap1 bound with all Sp1 truncated mutants, except for Sp1 ΔNTR, indicating that it interacts with the N-terminal region (NTR) domain of Sp1 (Fig. 4*B*). Further studies revealed that Sp1 bound to full-length Keap1, but not Keap1 ΔDGR (Fig. 4*C*), which is the Nrf2-binding domain.

### Ubiquitination of Sp1 by Keap1

**Figure 5:**
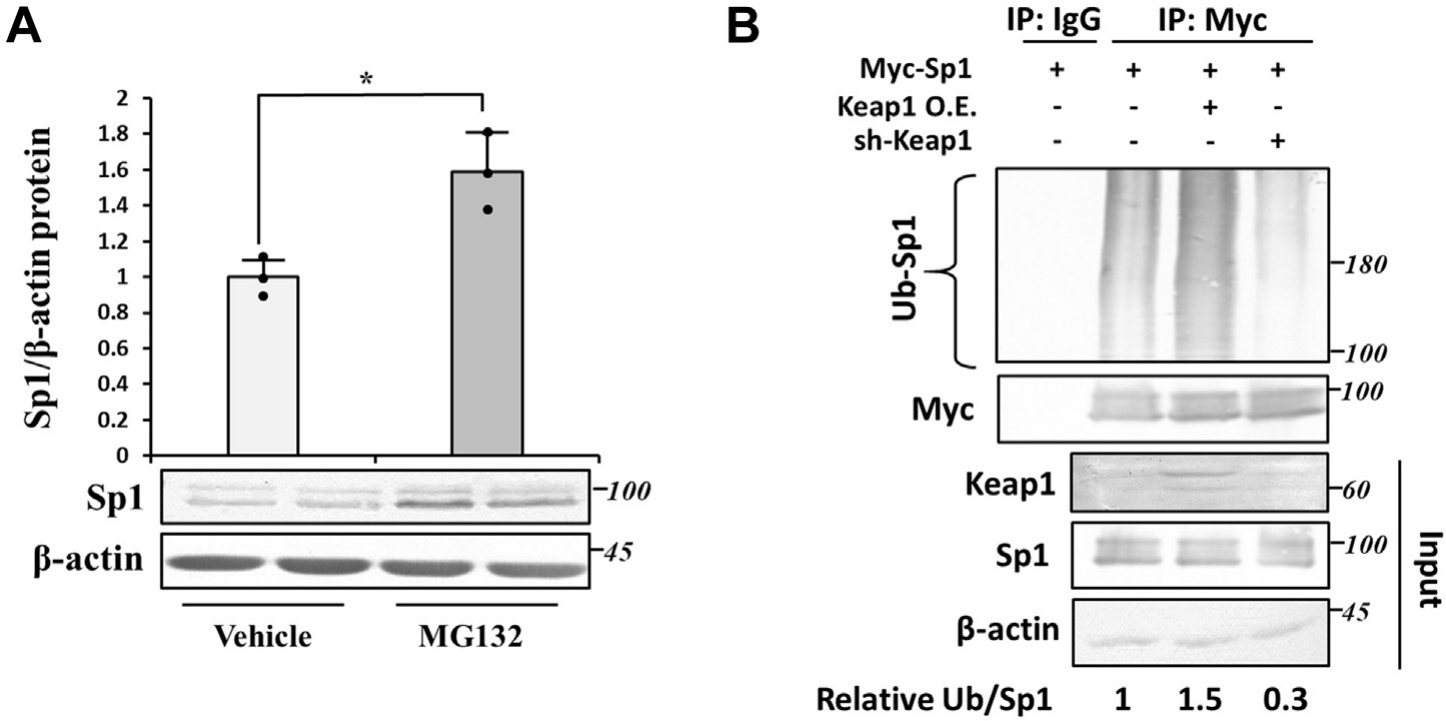
**Effects of Keap1 overexpression and KD on Sp1 ubiquitination.***A*, Hep3B cells were treated with the proteasome inhibitor MG132 (5 μM, 8 h), and Sp1 protein levels were measured by immunoblotting against an anti-Sp1 antibody. Protein loading was normalized by β-actin. The vehicle-treated cell value was set as 1.0. Graphs are means ± SD from three independent experiments. ∗*p* < 0.05. *B*, control (sh-GFP) or Keap1 KD (sh-Keap1) cells were transfected with pCMV-Sp1, pcDNA-Keap1, or both. Cells were grown in the presence of MG132 (5 μM, 8 h), cell lysates were immunoprecipitated using an anti-Myc antibody, and immunoblotting using anti-Myc, anti-ubiquitin, anti-Keap1, anti-Sp1 and anti-β-actin antibodies was then performed. The quantification of ubiquitinated Sp1 relative to immunoprecipitated Sp1 was performed using ImageJ. KD, knockdown; Keap1, Kelch-like ECH-associated protein 1; Sp1, specificity protein 1.

The direct interaction of Sp1 and Keap1 prompted us to hypothesize that Keap1 subjects Sp1 to ubiquitination because Keap1 is a substrate adaptor protein for the Cullin 3-containing E3 ubiquitin ligase complex (2). We then investigated whether Sp1 is regulated by ubiquitin-dependent proteasome degradation. Sp1 protein levels were elevated by the treatment with the proteasome inhibitor MG132 (5 μM, 8 h), confirming that Sp1 is subjected to proteasomal degradation (Fig. 5*A*). We then examined the ubiquitination of Sp1 by Keap1 in the presence of MG132. Sp1 incorporated approximately 1.5-fold more ubiquitin in Keap1-overexpressing cells than in control cells, and the KD of Keap1 significantly decreased (∼70%) the ubiquitination of Sp1 (Fig. 5*B*). Collectively, these results showed that Keap1 directly regulated the protein stability of Sp1 *via* the ubiquitin-dependent proteasome pathway.

### The role of Sp1 on Nrf2 protein levels

**Figure 6:**
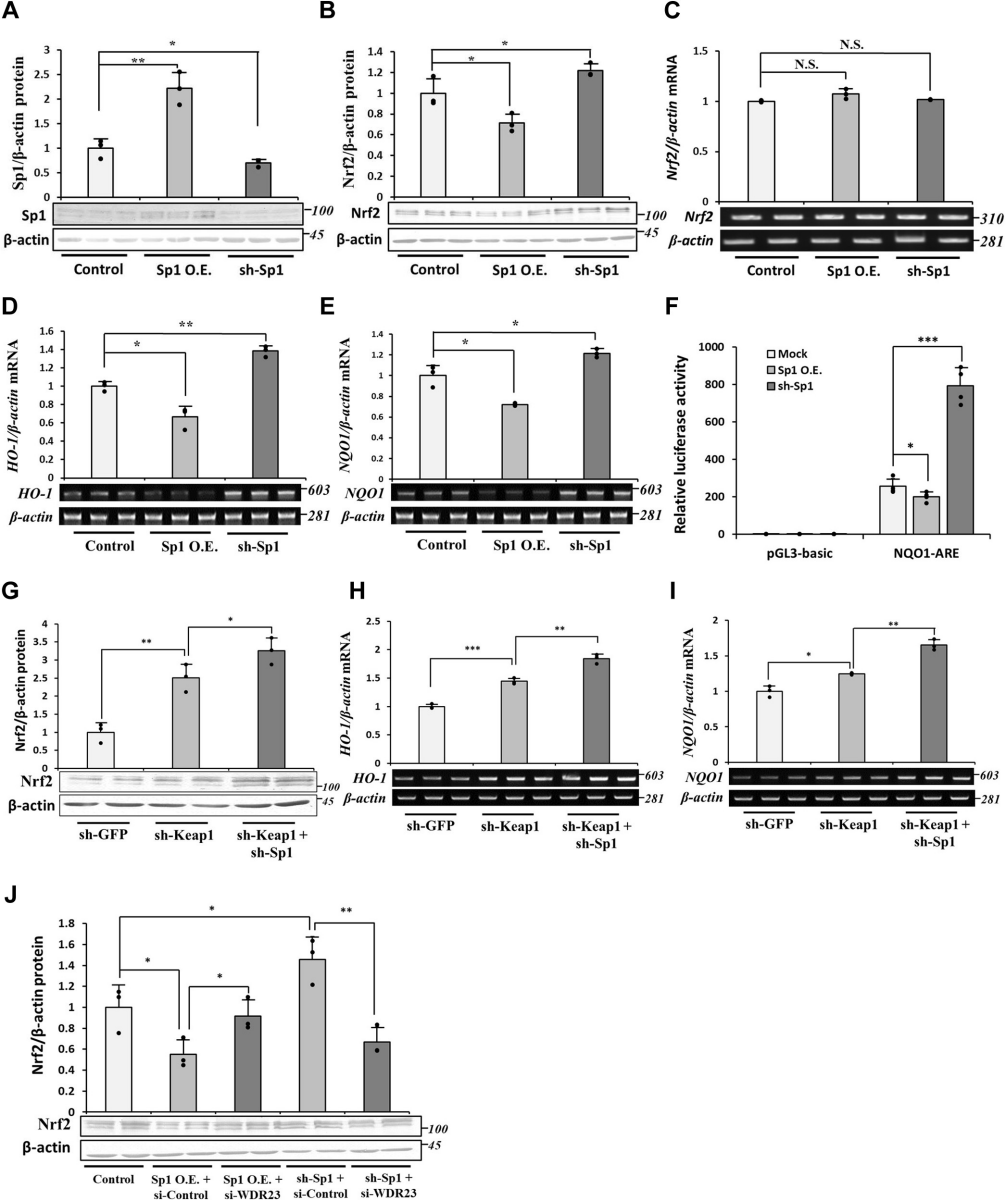
**Effects of Sp1 on Nrf2 protein levels.***A*–*F*, Sp1 knockdown cells were generated using sh-RNA and overexpression was performed by the transfection of Sp1 in pCMV-Myc. Control cells were sh-GFP + pCMV-Mock. *A*, knockdown and overexpression were confirmed by immunoblotting against an anti-Sp1 antibody. *B*, the abundance of intracellular Nrf2 was measured with immunoblotting against an anti-Nrf2 antibody. *C–E*, Nrf2, HO-1, and NQO1 mRNA levels were assessed in cell lysates by RT-PCR. *F*, Nrf2 activity was measured in cells expressing an NQO1-ARE luciferase reporter. Luciferase activity was measured 48-h posttransfection. Graphs are means ± SD from three independent experiments. *G*, the effects of Keap1 and Sp1 KD on Nrf2 protein abundance were observed by immunoblotting. *H* and *I*, the effects of Keap1 and Sp1 KD on the expression of HO-1 and NQO1 levels were assessed in cell lysates by RT-PCR. *J*, control (sh-GFP) or Keap1 KD (sh-Keap1) was co-transfected with pCMV-Mock or pCMV-Sp1 and si-Control or si-WDR23. The protein level of Nrf2 was then observed with immunoblotting. All graphs present means ± SD from three independent experiments. N.S. not significant, ∗*p* < 0.05, ∗∗*p* < 0.01, ∗∗∗*p* < 0.001 *versus* the indicated cells. CUL4A, Cullin4A; HO-1, heme oxygenase-1; Keap1, Kelch-like ECH-associated protein 1; NQO1, NAD(P)H quinone dehydrogenase 1; Nrf2, nuclear factor erythroid 2-related factor 2; Sp1, specificity protein 1.

The relationship between Sp1 and Keap1, in addition to the role of Sp1 in the regulation of *CUL4A* expression, prompted us to investigate whether Sp1 correlates with the intracellular level of Nrf2. Previous studies suggested that the protein level and transcriptional activity of Sp1 are induced by oxidative stress; however, their relationship with Nrf2 remains unclear (28, 29, 30). The overexpression and KD of Sp1 (Fig. 6*A*) decreased and increased Nrf2 protein levels, respectively (Fig. 6*B*). These effects of Sp1 on Nrf2 appeared to occur at the posttranscriptional level because the mRNA levels of Nrf2 were unaltered by the overexpression or KD of Sp1 (Fig. 6*C*). This is consistent with Sp1 being a mediator of the activation of the WDR23 pathway when the function of Keap1 is disabled. Next, the overexpression and KD of Sp1 decreased and increased Nrf2 transcriptional activity as shown by the assessment of Nrf2 target genes expression, the *HO-1* (Fig. 6*D*) and *NQO1* (Fig. 6*E*). These results were further supported by the evaluation of ARE luciferase reporter which showed that Nrf2 activity was inhibited and enhanced by overexpression and KD of Sp1, respectively (Fig. 6*F*). Furthermore, the KD of Sp1 further increased Nrf2 protein levels and transactivity in Keap1 KD cells (Fig. 6, *G*–*I*), which may be attributed to the inability of these cells to upregulate the expression of *CUL4A* and activate the WDR23 pathway (consistent with Fig. 2*L*). Nevertheless, this raises the critical question of whether Sp1 is really upstream of WDR23 activity or functions as an independent pathway for Nrf2 stability. Therefore, we treated Sp1-overexpressing and KD cells with siRNA against both isoforms of WDR23. The result obtained demonstrated that the KD of WDR23 attenuated the effects of Sp1 on Nrf2 (Fig. 6*J*), supporting our proposal that Sp1 requires and acts as an upstream regulator of WDR23. Collectively, these results highlight the relevance of Sp1 and the activity of the WDR23-dependent Nrf2 regulatory pathway.

### Sp1 or CUL4A overexpression recapitulates Keap1 KD required for WDR23 activity

**Figure 7:**
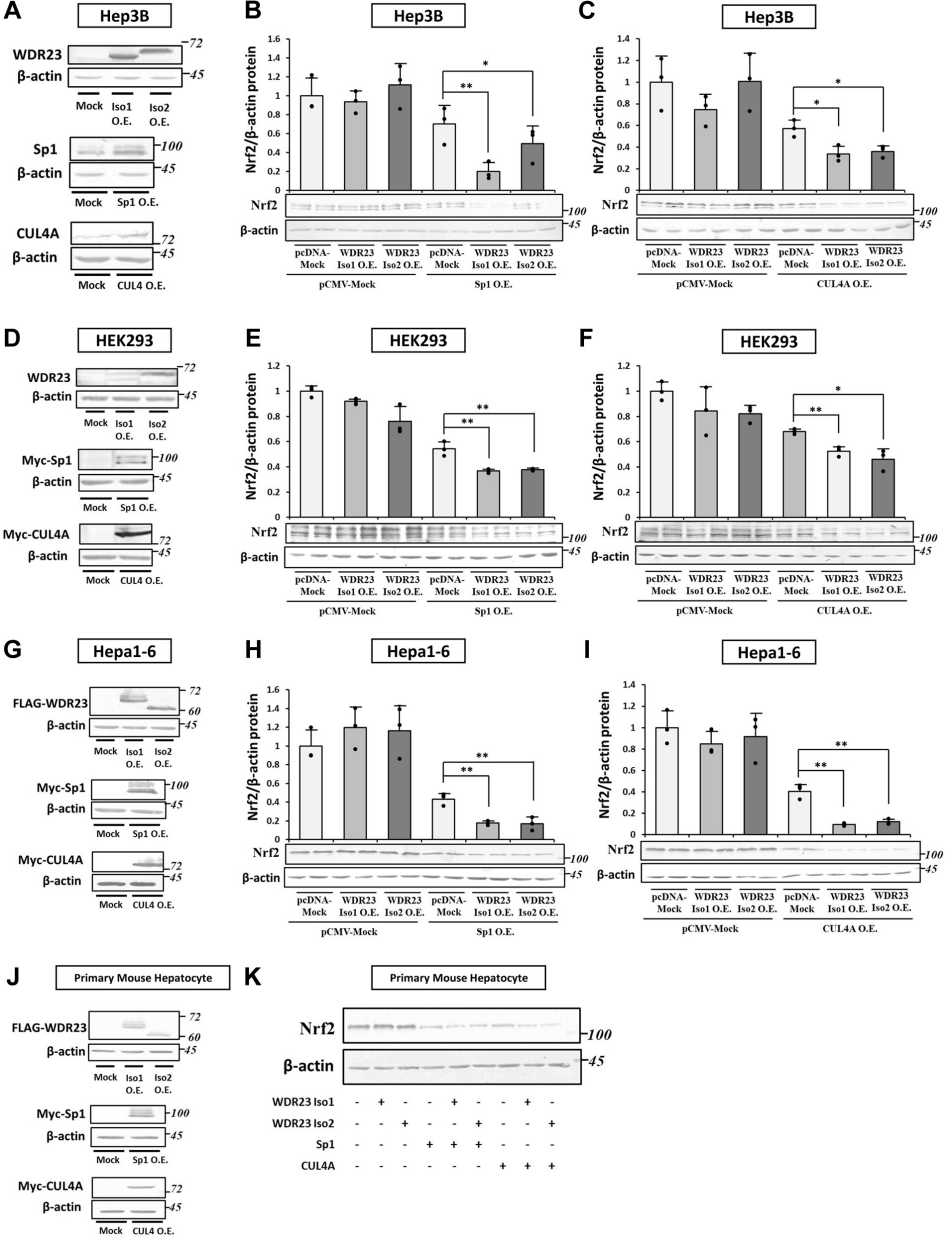
**Effects of Sp1 and CUL4A overexpression on the activity of ectopic WDR23**. *A–C*, WDR23 isoform 1 or 2 in 3×FLAG-pcDNA4 and Sp1 or CUL4A in pCMV-Myc were transfected into Hep3B cells. *A*, overexpression was confirmed by immunoblotting with an anti-WDR23, anti-Sp1, or anti-CUL4A antibody. *B* and *C*, immunoblotting analysis of Nrf2 levels with 15 μg of the total cell lysate from cells co-transfected with pcDNA-Mock, pcDNA-WDR23 isoform 1 or 2, and pCMV-Mock, pCMV-Sp1, or pCMV-CUL4A. *D–F*, the 3×FLAG-pcDNA4 vector containing WDR23 isoform 1 or 2 and pCMV-Myc containing Sp1 or CUL4A were transfected into HEK293 cells. *D*, overexpression was confirmed by immunoblotting. *E* and *F*, immunoblotting analysis of Nrf2 levels with 15 μg of the total cell lysate from cells co-transfected with pcDNA-Mock, pcDNA-WDR23 isoform 1 or 2, and pCMV-Mock, pCMV-Sp1 or pCMV-CUL4A. *G–I*, WDR23 isoform 1 or 2 in 3×FLAG-pcDNA4 and Sp1 or CUL4A in pCMV-Myc were transfected into HEPA1-6 cells. The abundance of WDR23, Sp1, CUL4A, and Nrf2 was measured with immunoblotting. *J* and *K*, WDR23 isoform 1 or 2 in 3×FLAG-pcDNA4 and Sp1 or CUL4A in pCMV-Myc were transfected into primary mouse hepatocyte cells. The abundance of WDR23, Sp1, CUL4A, and Nrf2 was assessed with immunoblotting. All graphs present means ± SD from three independent experiments. ∗*p* < 0.05, ∗∗*p* < 0.01 *versus* the indicated cells. CUL4A, Cullin4A; Nrf2, nuclear factor erythroid 2-related factor 2; Sp1, specificity protein 1; WDR23, WD40 repeat protein 23.

If Sp1-regulated *CUL4A* expression mediates crosstalk between Keap1 and WDR23, the direct overexpression of Sp1 or CUL4A is expected to bypass the requirement of the KD of Keap1 in cells to respond to the overexpression of WDR23. We used this strategy to further confirm whether Sp1 and CUL4A play critical roles in the crosstalk between the two independent and parallel regulators of Nrf2. The overexpression of both isoforms of WDR23, Sp1, and CUL4A in Hep3B cells was confirmed by immunoblotting (Fig. 7*A*). Consistent with earlier results, no changes were observed in total Nrf2 protein levels after the overexpression of WDR23 in control Hep3B cells. However, the co-expression of WDR23 with Sp1 markedly decreased Nrf2 protein levels by ∼80 and ∼50% for the overexpression of WDR23 isoforms 1 and 2, respectively (Fig. 7*B*). Furthermore, the co-expression of WDR23 with CUL4A also reduced Nrf2 protein levels (Fig. 7*C*). These results demonstrated that Sp1 and CUL4A play roles in and mediate the activation of the WDR23 pathway, particularly under Keap1 KD conditions. To study whether this phenomenon also occur in normal cells, we used a human embryonic kidney (HEK293) cells. We obtained similar results by using these cells (Fig. 7, *D*–*F*) suggesting that Sp1-CUL4A axis also plays role to mediate crosstalk between Keap1 and WDR23 pathways in normal cells and independent of the cell type. Next, we used murine hepatoma cell line, Hepa1-6 (Fig. 7, *G*–*I*). The results showed similar trend indicating that this mechanism is conserved among mammalians. In addition, we validated our finding by using primary mouse hepatocyte cells (Fig. 7, *J* and *K*) that provide more relevant and reflective results to that of the *in vivo* environment.

### Effects of the overexpression of CRL4A^WDR23^ on Nrf2 levels and activity

**Figure 8:**
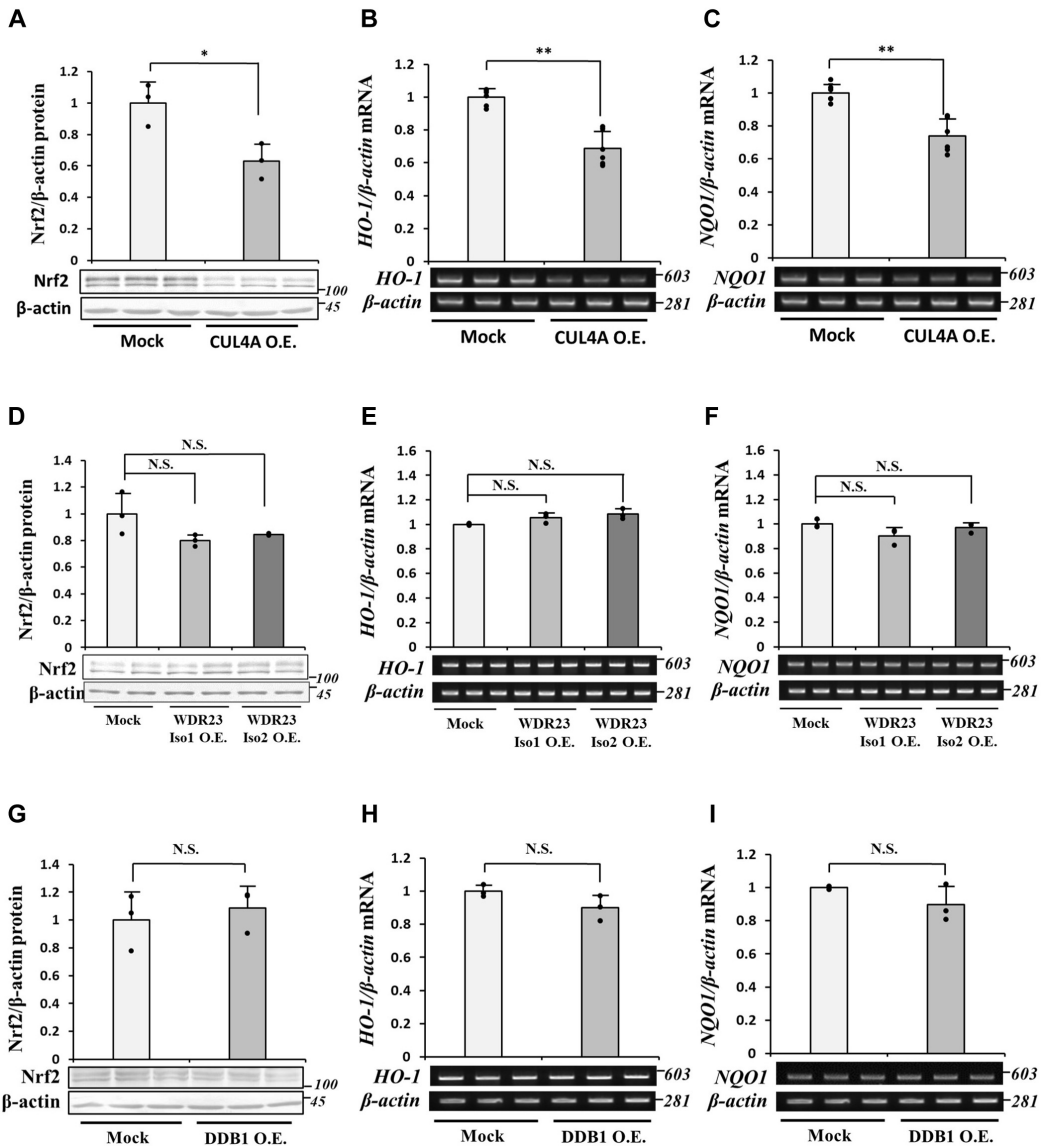
**Effects of CUL4A, WDR23, and DDB1 overexpression on Nrf2 levels and activity**. *A–C*, CUL4A in the pCMV-Myc vector was transfected into Hep3B cells. *A*, Nrf2 protein levels were analyzed by immunoblotting. *B* and *C*, the abundance of mRNA encoding HO-1 and NQO1 was assessed from Mock and CUL4A-overexpressing cells by RT-PCR. *D*, the abundance of Nrf2 in WDR23 isoforms 1 and 2 overexpressing Hep3B cells was examined by immunoblotting. *E* and *F*, the abundance HO-1 and NQO1 mRNA in WDR23 isoforms 1 and 2 overexpressing Hep3B cells was examined by RT-PCR. *G*, Nrf2 protein levels were assessed in cell lysates obtained from DDB1-overexpressed Hep3B cells. *H* and *I*, the abundance of mRNA encoding HO-1 and NQO1 was examined from mock and DDB1-overexpressed cells by RT-PCR. All graphs present means ± SD from three independent experiments. ∗*p* < 0.05, ∗∗*p* < 0.01 *versus* the indicated cells. CUL4A, Cullin4A; DDB1, DNA damage-binding protein 1; HO-1, heme oxygenase-1; NQO1, NAD(P)H quinone dehydrogenase 1; Nrf2, nuclear factor erythroid 2-related factor 2; WDR23, WD40 repeat protein 23.

The contribution of *CUL4A* gene expression on the regulation of CRL4A^WDR23^ activity toward Nrf2 suggesting that the CUL4A is the rate-limiting factor of this E3 ligase complex. To test this, we overexpressed individual component of CRL4A^WDR23^ complex. We found that the overexpression of CUL4A alone was sufficient to decrease Nrf2 protein levels (Fig. 8*A*) and downregulate the expression of Nrf2 target genes (Fig. 8, *B* and *C*). In contrast, the overexpression of WDR23 (Fig. 8, *D*–*F*) or DDB1 (Fig. 8, *G*–*I*) had no effects on both protein levels and activity of Nrf2. These results revealed that CUL4A is indeed the rate-limiting factor of CRL4A^WDR23^. We herein established that the Sp1-mediated upregulated expression of *CUL4A* during the KD of Keap1 is the mechanism underlying crosstalk between the Keap1 and WDR23 pathways for the regulation of Nrf2.

**Figure 9:**
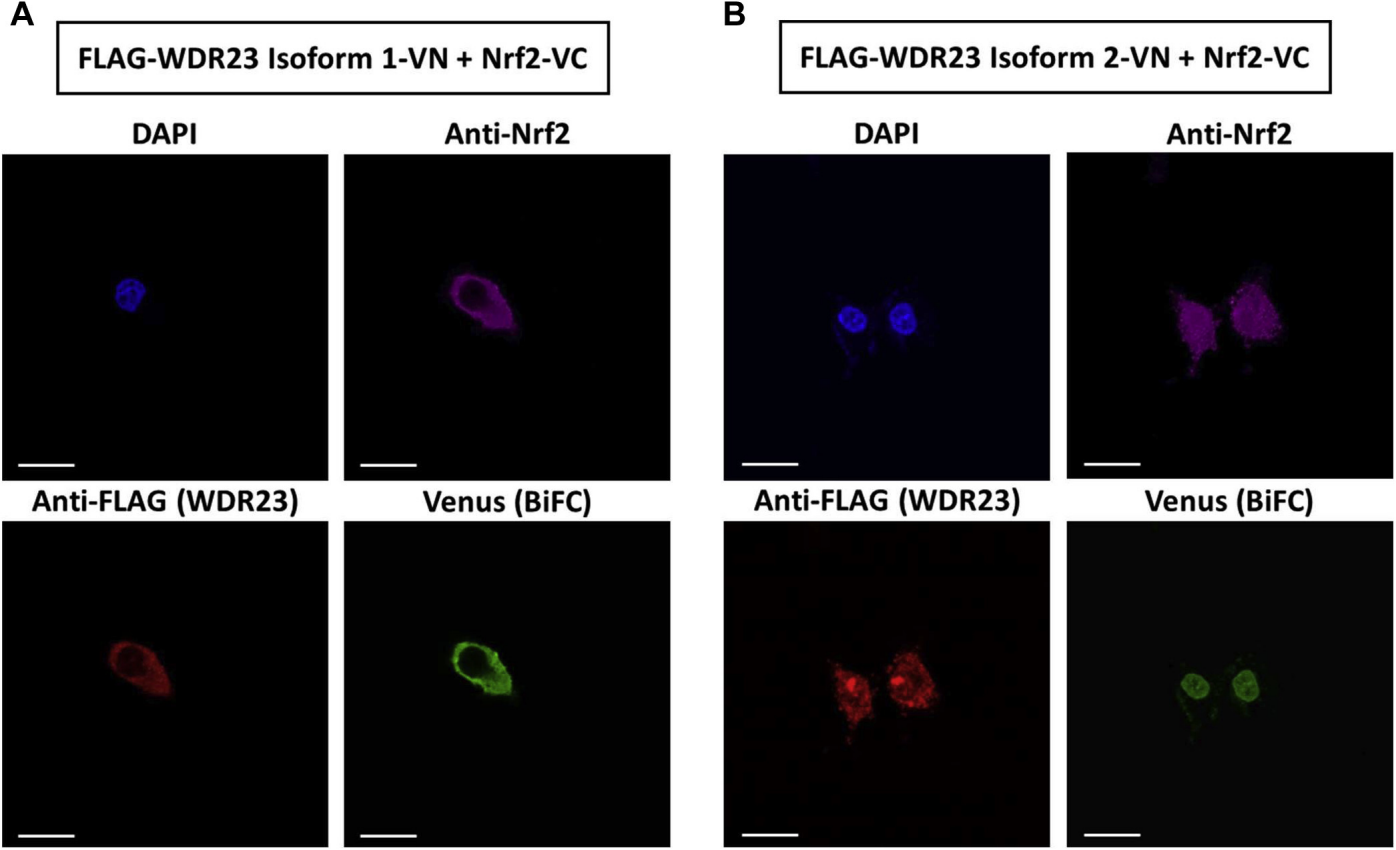
**Intracellular interaction of different isoforms of WDR23 with Nrf2.** N- and C-terminal fragments of Venus fluorescent proteins were fused to the C-terminal of FLAG-WDR23 isoform 1 or FLAG-WDR23 isoform 2 and Nrf2, respectively. The interaction between each isoform of WDR23 and Nrf2 brings the N- and C-terminal fragments in proximity to reconstitute an intact fluorescent protein. *A*, FLAG-WDR23 isoform 1-VN was co-expressed with Nrf2-VC in Hep3B. *B*, FLAG-WDR23 isoform 2-VN was co-expressed with Nrf2-VC in Hep3B cells. Overexpression was confirmed with anti-Nrf2 or anti-FLAG antibodies. Venus fluorescence as the BiFC signal indicates the interaction. Scale bar: 20 μm. BiFC, bimolecular fluorescence complementation; Nrf2, nuclear factor erythroid 2-related factor 2; WDR23, WD40 repeat protein 23.

We and others also demonstrated that WDR23 isoforms 1 and 2 primarily localized to the cytosol and nucleus, respectively, of cells in both humans (11, 12) and the nematode *Caenorhabditis elegans* (35). However, the subcellular localization pattern of the WDR23–Nrf2 complex, which impacts the biological importance of the regulation of Nrf2 by WDR23, remains unknown. In the present study, using the BiFC assay, we confirmed that the WDR23 isoform 1–Nrf2 complex was present in the cytosol, whereas the WDR23 isoform 2–Nrf2 complex was observed in the nucleus (Fig. 9). These results indicated that isoforms 1 and 2 of WDR23 regulate cytosolic and nuclear Nrf2, respectively.

## Discussion

**Figure 10:**
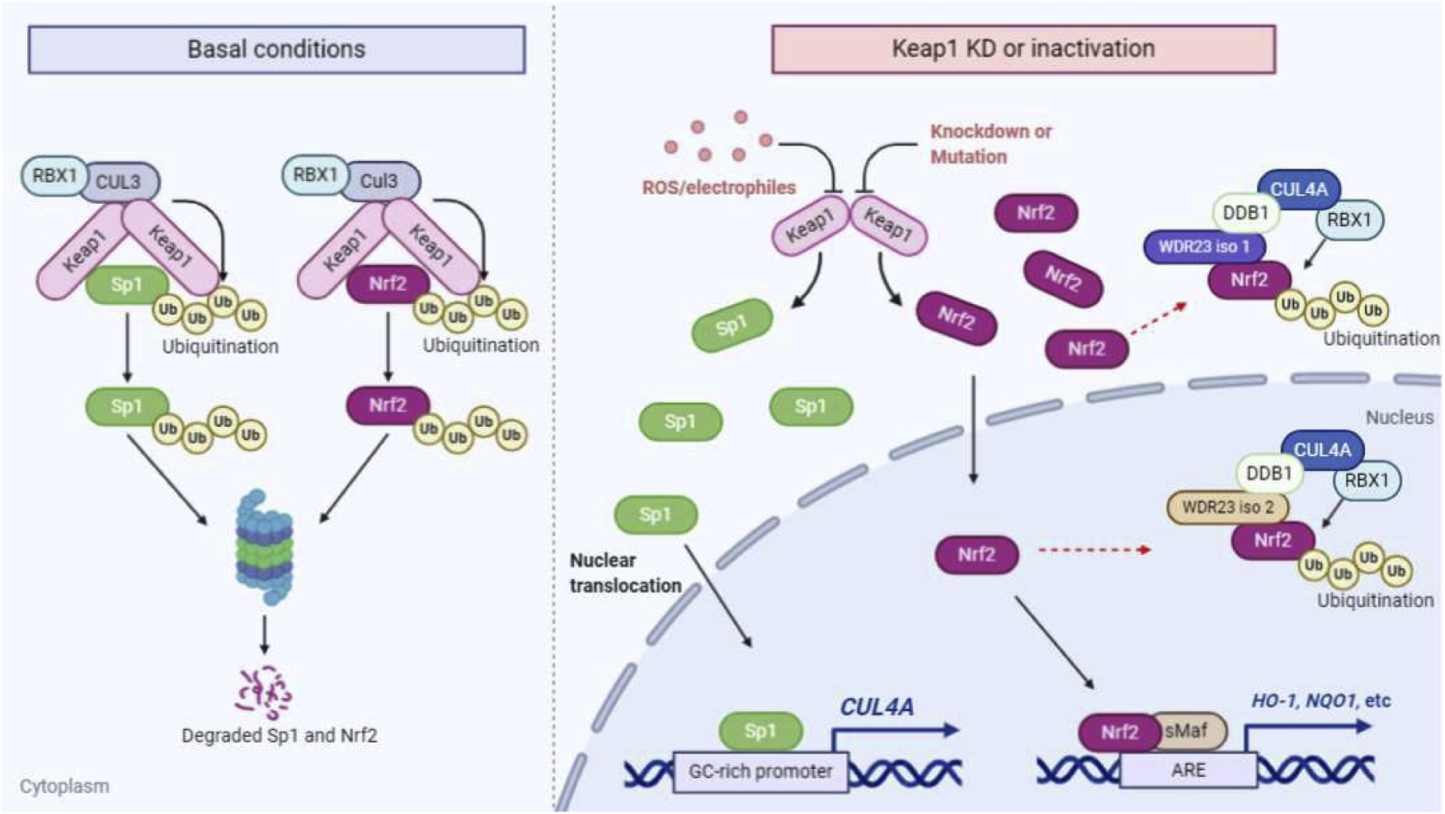
**Proposed model of crosstalk between Keap1 and WDR23 pathways in Nrf2 regulation.** Under basal conditions, Keap1 interacts with both Nrf2 and Sp1. These interactions lead to the ubiquitination and sequential degradation of Nrf2 and Sp1, maintaining their low basal protein levels. The downregulation or inactivation of Keap1 by oxidative stress or somatic mutations leads to the stabilization and transactivation of Nrf2 and Sp1. In this regard, accumulated Sp1 induces the transcription of *CUL4A*. Overexpressed CUL4A acts as a scaffold that associates with WDR23, DDB1, and RBX1 to form a functional CRL4A^WDR23^ E3 ligase that promotes the ubiquitination of Nrf2. CUL4A, Cullin4A; DDB1, DNA damage-binding protein 1; Keap1, Kelch-like ECH-associated protein 1; Nrf2, nuclear factor erythroid 2-related factor 2; RBX1, Ring-box 1; Sp1, specificity protein 1; WDR23, WD40 repeat protein 23.

The aberrant activation of Nrf2 is frequently observed in many cancers and promotes cancer growth and metastasis and also confers chemoresistance and radioresistance (36, 37, 38). Somatic mutations within Keap1 or Nrf2 (exclusively found in the DLG and ETGE motifs) are the most frequent causes of hyperactive Nrf2 in cancer (37). Therefore, determining other regulatory mechanisms of Nrf2 that may be used as a therapeutic target to improve cancer responses to chemotherapy are important. WDR23 was recently identified as a novel regulator of Nrf2 and Nrf2-dependent drug-metabolizing enzymes (11, 12). We previously reported that the WDR23 pathway was activated when the function of Keap1 was impaired; however, the underlying mechanisms remain unclear. In the present study, we elucidated the mechanism underlying the activation of WDR23 during the inhibition of Keap1, which was mediated by the Sp1-regulated expression of *CUL4A* (Fig. 10).

In the present study, we revealed that the expression of *CUL4A*, but not that of other components of CRL4A^WDR23^, was upregulated by the KD of Keap1. In an examination of the promoter region of *CUL4A*, we identified multiple Sp1 putative binding sites. The effects of the KD of Keap1 appear to require Sp1 because the mithramycin A and KD of Sp1 prevented the expression of CUL4A being upregulated by Keap1 KD. Based on the critical role of Sp1 in this context, we investigated the ability of Sp1 to regulate the expression of CUL4A. The KD of Keap1 and overexpression of Sp1 both induced the promoter activity of a 1920-bp fragment of the 5′-flanking sequence and its consecutive 5′-end deletion toward the −230 bp of *CUL4A*. There are four possible Sp1-binding sites within the −230/+50 region of *CUL4A*, and by using site-directed mutagenesis, we showed that the fourth site (−30/−21 bp from TSS; Sp1_M4) was the critical region for the regulation of *CUL4A* promoter activity by Sp1. Additionally, the ChIP assay revealed that Sp1 was associated with this binding site. The present study is the first to reveal a relationship between the transcription factor Sp1 and the regulation of *CUL4A* gene expression.

Despite its crucial role in malignancy, the regulation of CUL4A at the transcriptional level has not yet been examined in detail. For example, a study on the Wnt-dependent regulation of p27^KIP1^ unexpectedly showed that the TCF/LEF1 complex bound to and activated the promoter of mouse *Cul4a* (39). A recent study reported that cyclic adenosine monophosphate response element–binding protein (CREB) bound to the cyclic adenosine monophosphate responsive element located in the −926/−764 of *CUL4A* and activated its transcription (40). The present results revealed that the −230 bp fragment of the 5′-flanking sequence of *CUL4A* did not contain TCF/LEF1 or CREB-binding motifs but was still responsive to the KD of Keap1, indicating that neither the Wnt pathway nor CREB are involved in this context.

Sp1 is a crucial transcriptional factor that regulates the basal transcription of genes with a TATA-less promoter. Deniaud *et al.* (41) identified a set of genes that are regulated by Sp1 using genome-wide expression profiling, including those involved in metabolism, the transcriptional machinery, adhesion, apoptosis, cell growth, exocytosis, inflammation, signal transduction, ubiquitination, and many genes with unknown functions (41). Regarding the role of Sp1 in the expression of ubiquitination-related factors, they found that the overexpression of Sp1 upregulated the expression of ariadne E2-binding protein homolog 1 and F-box and WD-40 domain protein 2 and downregulated the expression of praja 2 E3 ligase. Burger *et al.* (42) indicated that Sp1 regulated the basal transcriptional activity of Breast Cancer-Associated gene 2 E3 ligase (42). In the present study, the result showing that Sp1 regulated the basal and inducible expression of *CUL4A* expands our knowledge on the involvement of Sp1 as a regulator of the ubiquitination pathway.

The present results demonstrated that the increase observed in Sp1 following the KD of Keap1 was because of the direct regulation of Sp1 by Keap1. We showed that the DGR domain of Keap1 physically interacted with the NTR domain of Sp1 and subjected Sp1 to ubiquitination. Therefore, the KD of Keap1 was directly responsible for the stabilization of Sp1 and its transcriptional activity. Keap1 binds to conserved DLG and ETGE in Nrf2; however, the NTR domain of Sp1 does not contain these motifs. Instead, we identified the DLT motif within the NTR domain of Sp1, which resembled the DLG domain of Nrf2. Similar to this result, iASPP was previously shown to interact with Keap1 through its DLT motif (43), suggesting that Sp1 binds with Keap1 *via* this DLT motif.

The regulation of Sp1 by ubiquitination has not yet been examined in detail. To the best of our knowledge, only two proteasome-mediated degradation pathways have been shown to regulate Sp1. β-transducin repeat-containing protein subjects Sp1 to proteasomal degradation in response to glucose starvation (44), and Ring finger protein 4 targets sumoylated Sp1 for degradation under basal conditions (45, 46). In the present study, we revealed the role of Keap1 as a novel regulator of Sp1 ubiquitination and proteasomal degradation, which provides additional insights into alternative mechanisms of Sp1 activation under oxidative stress conditions.

The direct Keap1–Sp1 interaction implies that structural changes in Keap1 by reactive oxygen species and electrophiles may also induce the stabilization of Sp1, similar to Nrf2. For example, hydrogen peroxide has been shown to modify four cysteine residues (Cys226, Cys613, Cys622, and Cys624) in Keap1, thereby inducing structural modifications (47). Consistent with these findings, Ryu *et al*. (28) were the first to report that oxidative stress increases Sp1 protein levels and transactivity in neurons. We also previously demonstrated that high glucose-induced oxidative stress increased nuclear Sp1 levels (27). However, Yeh *et al.* (48) showed that H_2_O_2_-induced Sp1 protein levels were mediated by the activation of the internal ribosomal entry site pathway and increased the translation of Sp1, suggesting the Keap1-independent regulation of Sp1 by oxidative stress (48). Collectively, the present results and previous findings imply the multilayered, involving translational and posttranslational, regulation of Sp1 by oxidative stress.

A previous study reported that Sp1 regulated the expression of *Keap1* by directly binding to its binding sites in the −160/−153 region of the *Keap1* promoter (49). These findings combined with the present results suggest that the regulation of Sp1 by Keap1 provides an autoregulatory feedback loop that compensates for reduced Keap1 activity. This positive feedback of Keap1 gene expression and the additional induction of the CRL4A^WDR23^ pathway may ensure a robust and efficient response toward insults on the Keap1-dependent Nrf2 regulation pathway.

Growing evidence has shown that Sp1 and CUL4A are both overexpressed in many cancers and are associated with a poor prognosis (50, 51, 52, 53). The mechanisms contributing to high Sp1 protein levels in tumors remain unclear (51); therefore, the present results indicate that the somatic mutations in *Keap1* frequently observed in various tumors are the reason for elevated Sp1 protein levels. Additionally, the targeting of Sp1 as a cancer treatment has been suggested (50); however, the role of Sp1 in malignancy is complex. Sp1 activates and suppresses the expression of oncogenes and tumor suppressor genes as well as genes involved in essential cellular functions (54). In the present study, the activation of Sp1 under Keap1 KD conditions was important for the activation of the CRL4A^WDR23^ machinery and prevented the constitutive activation of Nrf2, which may enhance the expression of drug-metabolizing enzymes. Based on the results of the present study, we added a role for Sp1 as a tumor suppressor factor. A more comprehensive understanding of the function of Sp1 in cancer is needed to verify its potential as a therapeutic target.

In the present study, the overexpression of both Sp1 and CUL4A may recapitulate the necessity of the KD of Keap1 for the activity of ectopically expressed WDR23 on Nrf2, which further supports the concept that Sp1 and CUL4A mediate the interplay between Keap1 and WDR23. It is important to note that the overexpression of CUL4A alone was sufficient to decrease Nrf2 protein levels, indicating that CUL4A is the rate-limiting factor of CRL4A^WDR23^ under basal conditions. Therefore, the upregulated expression of CUL4A during the KD of Keap1 is the mechanism underlying the crosstalk between the Keap1 and WDR23 pathways for the regulation of Nrf2. Research has so far focused on the role of CUL4A in the regulation of the cell cycle and maintenance of genomic integrity. Hence, the present results provide a novel insight into the contribution of inducible CUL4A expression in the oxidative stress response and Nrf2-dependent drug metabolism.

In conclusion, we herein identified a novel role for Keap1 as a regulator of Sp1 stability. We established that Sp1 is a transcriptional activator of *CUL4A*. Furthermore, *CUL4A* appears to be the rate-limiting factor of CRL4A^WDR23^. Collectively, the present results revealed that during the KD or inactivation of Keap1, Sp1 evades proteasomal degradation and activates the transcription of *CUL4A*, which ultimately leads to the activation of the CRL4A^WDR23^ machinery regulating the protein levels of Nrf2. The present study elucidated the molecular mechanism underlying the crosstalk between two independent and parallel regulators of Nrf2, which may be useful for the development of a therapeutic strategy for Nrf2-dependent cancer chemoresistance.

## Experimental procedures

### Materials

Dulbecco's modified Eagle's medium, tBHQ, hydrogen peroxide, an anti-DYKDDDDK (FLAG) antibody, anti-Myc tag monoclonal antibody, anti-GFP(VC) antibody (mFX75), and horseradish-peroxidase-conjugated goat anti-mouse IgG were purchased from Wako Pure Chemical Industries. MG132 (Z-Leu-Leu-Leu-H) was purchased from the Peptide Institute. Mithramycin A was from Cayman Chemicals. Penicillin-streptomycin solution, fetal bovine serum, and geneticin (G418) were from Sigma Chemical Co. Nitrocellulose membrane, horseradish-peroxidase-conjugated goat anti-rabbit IgG, and 4-chloro-1-naphthol were purchased from Bio-Rad Laboratories. Isogen was from Nippon Gene, and Revert Aid M-MuLV Reverse Transcriptase was from MBI Fermentas. KOD Plus Neo and Fx Neo DNA polymerase were from Toyobo. DAPI (4′,6-diamidino-2-phenylindole) was from Dojindo. Alexa Fluor 594-conjugated goat anti-mouse IgG and Alexa Fluor 647-conjugated goat anti-rabbit IgG were from Abcam. Anti-CUL4, anti-KLF15, and anti-AP2α antibodies were from Santa Cruz Biotechnology. An anti-Sp1 antibody was purchased from Cell Signaling Technology. An anti-ubiquitin antibody (clone FK2) was from StressMarq Bioscience. The anti-Nrf2, anti-Keap1, anti-WDR23, and anti-β-actin antibodies were prepared as described previously (12, 55).

### Plasmid constructs

The entire coding region of human Sp1 (GenBank accession number NM_138473.2) was amplified by PCR with primer sets 1 and 2 (Table 1). Primers were accompanied by a restriction site (underline). Amplified DNA was then digested by the restriction enzymes *EcoR*I and *Xho*I and ligated into the pCMV-Myc vector (Clontech Laboratories). In the BiFC assay, Sp1 cDNA was amplified with primers 2 and 3, VN155 without the stop codon was amplified using pBiFC-VN155 (Addgene) as a template with primer sets 4 and 5, and oligonucleotide sets 6 and 7 for the linker (GGGS)_2_ were annealed and then subsequently inserted into the pcDNA3.1(+) vector (Invitrogen) with *EcoR*I and *Xho*I, *Hind*III and *Bam*HI, and *EcoR*I respectively. Mouse Sp1 (GenBank accession number AF022363.1) was amplified by PCR with primer sets 8 and 9, digested by the restriction enzymes *Sal*I and *Not*I, and ligated into the pCMV-Myc vector. The cDNA of human CUL4A (GenBank accession number NM_001008895.4) and mouse CUL4A (GenBank accession number NM_146207.3) were amplified by PCR with primers 10 and 11 and 12 and 13, respectively, and then inserted into the pCMV-Myc vector with the *Sal*I and *Not*I sites. The cDNA of human WDR23 isoform 1 (GenBank accession number NM_025230.4) was amplified using primer sets 14 and 15. The cDNA of human WDR23 isoform 2 (GenBank accession number NM_181357.2) was obtained by PCR with two steps using human WDR23 isoform 1 cDNA as a template, as previously described (12). In brief, nucleotide fragment 1 was amplified with primer sets 14 and 16. Nucleotide fragment 2 was amplified with primers 15 and 17. Full-length human WDR23 isoform 2 was amplified in the second round of PCR from fragments 1 and 2 with primers 14 and 15. Amplified human WDR23 isoforms 1 and 2 were digested by the restriction enzymes *Not*I and *Xba*I and then ligated into the 3×FLAG-pcDNA4 vector (Invitrogen). Mouse WDR23 isoform 1 (GenBank accession number NM_001199009.1) and isoform 2 (GenBank accession number XM_006519087.5) were amplified by PCR with primers 18 and 20 and 19 and 20, respectively, and then inserted the 3×FLAG-pcDNA4 vector with the *EcoR*I and *Not*I sites. Regarding BiFC, both isoforms of human WDR23 were amplified by PCR with primer sets 21 and 22 using pcDNA4/WDR23 isoforms 1 and 2 as templates, digested with *SalI* and *NotI*, and inserted into the pBiFC-VN155 vector. Human Keap1 cDNA (GenBank accession number NM_203500.2) was amplified by PCR with primers 23 and 24 and then inserted into the 3×FLAG-pcDNA4 vector with the *BamH*I and *Xho*I sites. In the BiFC assay, human Keap1 cDNA without a stop codon was amplified using primers 25 and 26 and then inserted into the pBiFC-VC155 vector (Addgene) with the *Sal*I and *Kpn*I sites. Human Nrf2 cDNA (GenBank accession number NM_006164.5) was amplified by PCR with primers 27 and 28 and then inserted into the pBiFC-VC155 vector with *Sal*I and *Kpn*I.

**Table 1:** Primers used for plasmid constructs

No.	Sequences	Descriptions
1	5′-CTGAATTCCCATGAGCGACCAAGATCACTCCAT-3′	Fw; 1–23 of human *Sp1* cDNA; underline, *EcoR*I site; double underline, start codon
2	5′-TAACTCGAGGTATGGCCCATATGTCTCTGGCC-3′	Rv; downstream of human *Sp1* cDNA; underline, *Xho*I site; stop codon is upstream of this primer
3	5′-GGAATTCATGAGCGACCAAGATCACTC-3′	Fw; 1–20 of human *Sp1* cDNA; underline, *EcoR*I site; double underline, start codon
4	5′-TATAAGCTTACCATGGTGAGCAAGGGCGAGGAGC-3′	Fw; 1–22 of VN155; underline, *Hind*III site; double underline, start codon
5	5′-TTGGATCCGGCGGTGAGATAGACGTTGTGGCTG-3′	Rv; 441–465 of VN155; underline, *BamH*I site
6	5′-AATTCTGGAGGTGGCGGGAGCGGAGGTGGCGGGAGTG-3′	(GGGS)_2_ linker sequence; sense
7	5′-AATTCACTCCCGCCACCTCCGCTCCCGCCACCTCCAG-3′	(GGGS)_2_ linker sequence; antisense
8	5′-ATAAGTCGACCATGAGCGACCAAGATCACTCCAT-3′	Fw; 1–23 of mouse *Sp1* cDNA; underline, *Sal*I site; double underline, start codon
9	5′-TAGCATTTATGCGGCCGCTTAGAAACCATTGCCACTGA-3′	Rv; 2336–2355 of mouse *Sp1* cDNA; underline, *Not*I site; double underline, start codon
10	5′-ATAAGTCGACCGCGGACGAGGCCCCGCGGAA-3′	Fw; 3–23 of human *CUL4A* cDNA; underline, *Sal*I site
11	5′-TAGCATTTATGCGGCCGCTCAGGCCACGTAGTGGTACT-3′	Rv; 2261–2280 of human *CUL4A* cDNA; underline, *Not*I site; double underline, stop codon
12	5′-ATAAGTCGACCATGGCGGACGAGGGCCCTCG-3′	Fw; 1–20 of mouse *CUL4A* cDNA; underline, *Sal*I site; double underline, start codon
13	5′-TAGCATTTATGCGGCCGCTCATGCCACGTAGTGGTACT-3′	Rv; 2261–2280 of mouse *CUL4A* cDNA; underline, *Not*I site; double underline, stop codon
14	5′-ATAAGAATGCGGCCGCATGGGATCGCGGAACAGCAG-3′	Fw; 1–20 of human *WDR23* isoform 1 cDNA; underline, *Not*I site; double underline, start codon
15	5′-TATCTAGACTACTGGGGTGAGGAAAAGG-3′	Rv; 1621–1641 of human *WDR23* isoform 1 cDNA; underline, *Xba*I site; double underline, stop codon
16	5′-GTCCAAGAGGGCCTGGGCCAGATCCACATC-3′	Rv; 115–144 of human *WDR23* isoform 2 cDNA; dotted underline, complementary to the primer 17
17	5′-CAGGCCCTCTTGGACTCAGA-3′	Fw; 129–149 of human *WDR23* isoform 2 cDNA; dotted underline, complementary to the primer 16
18	5′-CTGAATTCTATGGGATCACGGAACAGCAG-3′	Fw; 1–20 of mouse *WDR23* isoform 1 cDNA; underline, *EcoR*I site; double underline, start codon
19	5′-CTGAATTCTATGAAGATGTGGATCTGGCCCAGAGGCCAAGTGAGACTGG-3′	Fw; 1–40 of mouse *WDR23* isoform 2 cDNA; underline, *EcoR*I site; double underline, start codon
20	5′-TAGCATTTATGCGGCCGCCTACTGAGGTGAGGAAAAGG-3′	Rv; 1631–1650 of mouse *WDR23* isoform 1 cDNA; underline, *Not*I site; double underline, stop codon
21	5′-ATTAGTCGACTATGGATTACAAGGATGACGATGACAAG-3′	Fw; 1014–1037 of pcDNA4 TO-3×FLAG vector; underline, *Sal*I site; double underline, start codon
22	5′-AATGGTACCCTGGGGTGAGGAAAAGGGTG-3′	Rv; 1618–1638 of human *WDR23* isoform 1 cDNA; underline, *Kpn*I site.
23	5′-AATGGATCCATGCAGCCAGATCCCAGGCC-3′	Fw; 1–20 of human *Keap1* cDNA; underline, *BamH*I site; double underline, start codon
24	5′-CCGCTCGAGTCAACAGGTACAGTTCTGCT-3′	Rv; 1856–1875 of human *Keap1* cDNA; underline, *Xho*I site; double underline, stop codon
25	5′-ATTAGTCGACTATGCAGCCAGATCCCAGGCC-3′	Fw; 1–20 of human *Keap1* cDNA; underline, *Sal*I site; double underline, start codon
26	5′-CCGGGTACCACAGGTACAGTTCTGCTGGT-3′	Rv; 1853–1872 of human *Keap1* cDNA; underline, *Kpn*I site
27	5′-ATTAGTCGACTATGATGGACTTGGAGCTGCC-3′	Fw; 1–20 of human *Nrf2* cDNA; underline, *Sal*I site; double underline, start codon
28	5′-CCGGGTACCGTTTTTCTTAACATCTGGCT-3′′	Rv; 1796–1815 of human *Nrf2* cDNA; underline, *Kpn*I site

### RNA interference

Regarding the KD of Keap1 and Sp1 using shRNA, specific target regions of Keap1 and Sp1 were designated and inserted into the pBAsi-hU6 Neo Vector (Takara Bio Inc) according to a previously described procedure (27, 55). The target sequence for Keap1 KD was 5′-GCAGGCCTTTGGCATCATGAACG-3′ and the target for Sp1 was 5′-AATGCCAATAGCTACTCAACT-3′. The nucleotide sequence for control shRNA against GFP was 5′-CTCGAGTACAACTATAACTCA-3′. In the KD of WDR23, siRNA against WDR23 (Cat. No. SI05029899) with the target sequence of 5′-CUGGGUCUUUAGGGUAGGACA-3′ was purchased from Qiagen. AllStars Negative Control (SI03650318, Qiagen) was used as control siRNA. shRNA and siRNA were transfected into cells using ScreenFect A (Wako) according to the manufacturer's instructions. Transfectants of shRNA-Mock, shRNA-Keap1, or shRNA-Sp1 were selected using G418.

### Cell culture, transfection, and treatment

The human hepatoma cell line Hep3B was obtained from the Cell Resource Center for Biomedical Research at the Institute of Development, Aging, and Cancer of Tohoku University. The mouse hepatoma cell line Hepa1-6 (RCB1638) was from RIKEN BRC Cell Bank. Primary mouse hepatocyte was isolated from male C57BL/6JJc1 mice (5 weeks old, CLEA Japan) by the two-step collagenase perfusion technique according to previously described method with minor modifications (56). Experiments that include animals were conducted in accordance with the guidelines on the welfare of experimental animal and with approval of the Ethics Committee on the use of animals of Kwansei Gakuin University. Cells were cultured in Dulbecco's modified Eagle's medium containing 10% (v/v) fetal bovine serum, penicillin (100 units/ml), and streptomycin (100 μg/ml), and maintained at 37 °C in 5% CO_2_ and 95% air. The transfection of the indicated constructs was performed using the calcium phosphate method or Effectene (Qiagen). Cells were cultured in the presence or absence of mithramycin A (100 nM, 8 h), tBHQ (60 μM, 8 h), H_2_O_2_ (100 μM; 8 h), or MG132 (5 μM, 8 h).

### Immunoprecipitation and immunoblotting

Cells were washed with ice-cold PBS, collected, lysed in immunoprecipitation buffer (50 mM Tris-HCl, pH 8.0 and 150 mM NaCl, 1% Triton X-100, and 1 mM phenylmethylsulfonyl fluoride), and centrifuged at 14,000*g* for 15 min. The protein-containing supernatant was incubated with 2 μl of the anti-Myc antibody, anti-FLAG antibody, or unimmunized mouse serum at 4 °C for 2 h. Twenty microliters of protein G-Sepharose (50% (w/v); GE Healthcare) was then added to the solution and incubated at 4 °C for 1 h. Samples were washed with immunoprecipitation buffer containing 0.1% Triton X-100. Anti-Nrf2, anti-CUL4, anti-Sp1, anti-Keap1, anti-WDR23, anti-ubiquitin, anti-Myc, anti-FLAG, or anti-β-actin was used for immunoblotting. In the present study, β-actin was used as a loading control. Band intensity was quantified using NIH Image software ImageJ.

### BiFC assay

VN155 (N-terminal half of Venus) fused to the N terminal of Sp1 was co-expressed with VC155 (the C-terminal half of Venus) fused to the C terminal of Keap1. VN155 fused to the C-terminal of FLAG-WDR23 isoforms 1 and 2 was co-expressed with VC155 fused to the C terminal of Nrf2 in Hep3B cells. Twenty-four hours after transfection, cells were rinsed with PBS solution and fixed for 20 min with 4% paraformaldehyde in PBS, then rinsed with TPBS [PBS +0.2% Tween 20 (Bio-Rad)], followed by blocking with 0.1% bovine serum albumin (Wako) in TPBS. Cells were subsequently incubated either with the anti-Keap1 and anti-VC antibody or anti-FLAG and anti-Nrf2 antibodies, followed by an incubation with Alexa Fluor 594-conjugated goat anti-mouse IgG and Alexa Fluor 647-conjugated goat anti-rabbit IgG. The nucleus was counterstained using DAPI. Images were obtained by confocal microscopy TCS SP8 (Leica Microsystems). Venus fluorescence was detected as a BiFC signal.

### Luciferase reporter gene assay

Hep3B cells were transiently transfected with 0.25 μg of the pGL3-containing *CUL4A* promoter or NQO1-ARE and pRL-null vector (12.5 ng) as an internal control with GenePORTER TM2 transfection reagent (Gene Therapy Systems). Cells were co-transfected with 0.25 μg of shGFP, shKeap1, or shSp1 in pBAsi-hU6, pCMV-Mock, or pCMV-Sp1. Forty-eight hours posttransfection, luciferase activity was assayed with a luminometer (Lumat LB9507; Berthold) using the Dual-Luciferase Reporter Assay System (Promega) according to the manufacturer's protocol. Firefly luciferase activity was normalized to Renilla luciferase activity.

### ChIP assay

The ChIP assay was performed as described previously (27). In brief, Hep3B cells were crosslinked with 1.5% (w/v) formaldehyde for 10 min. The crosslinking reaction was quenched by the addition of glycine to a final concentration of 0.125 M. Cells were then washed three times with ice-cold PBS and lysed in ChIP buffer containing 0.5% NP-40, 1% Triton X-100, 150 mM NaCl, 50 mM Tris-HCl (pH 7.5), 0.5 mM DTT, 5 mM EDTA, 0.5 mM PMSF, and 10 mM NaF, sonicated on ice, and centrifuged (14,000*g* at 4 °C for 10 min). Fifty microliters of the supernatant was collected as the input, and the remnant was incubated with control IgG or the anti-Sp1 antibody at room temperature for 30 min. Protein A-Sepharose beads were added and incubated at 4 °C for 45 min. Beads were washed five times with ChIP buffer. After an extensive wash step, complexes were eluted with buffer containing 0.1 M NaHCO_3_ and 1% SDS followed by an incubation at room temperature for 15 min. Reverse crosslinking was performed with the addition of 0.4 M NaCl at 65 °C overnight. The mixture was then treated with proteinase K at 50 °C for 30 min, and DNA was purified. The *CUL4A* promoter fragments, −55 to +155, −265 to −56, and −825 to −626, were detected with primer sets 1 and 2, 3 and 4, and 5 and 6, respectively (Table 2).

**Table 2:** Primers used for ChIP assay

Primer number	Sequences	Descriptions
1	5′-CGGAGCTCGGCGGGCGGCGG-3′	Fw; upstream of *CUL4A* −55 to −36
2	5′-GGTCTGTCTCGGAAGTTCTT-3′	Rv; upstream of *CUL4A* +136 to +155
3	5′-CGGGAGTCCCGGCGCGCGCC-3′	Fw; upstream of *CUL4A* −265 to −246
4	5′-CTCCGCCCGCCCCGGTCCGC-3′	Rv; upstream of *CUL4A* −75 to −56
5	5′-TGAGGGGGCCCGGGGTCTTT-3′	Fw; upstream of *CUL4A* −825 to −806
6	5′-GCGCGGAGGGTCCTCCGCGG-3′	Rv; upstream of *CUL4A* −645 to −626

### Isolation of RNA and RT-PCR

Total RNA was extracted from cells using Isogen following the manufacturer's instructions and converted to cDNA by reverse transcription. PCR was performed with 10 pmol of each primer, Go Taq polymerase (Promega), and cDNA (100 ng) under the following conditions: 2 min at 94 °C and then a number of cycles at 94 °C for 30 s, 55 °C for 30 s, and 72 °C for 30 s. Primers, GenBank accession numbers, and sequences for PCR are listed in Table 3. PCR products were separated by electrophoresis on a 1% agarose gel, visualized with ethidium bromide staining, and quantified by scanning densitometry using ImageJ software (Version 1.36b; National Institutes of Health).

**Table 3:** Primers used for gene expression assessment

Primers	GenBank accession no.	Sequences	
Keap1	NM_203500.2	Forward	5′-TCTTCAAGGCCATGTTCACC-3′
		Reverse	5′-GGCACGCTGGTGCAACTCCA-3′
WDR23	NM_181357.2	Forward	5′-CACAGGATTGGAGAAGGAGG-3′
		Reverse	5′-TCGGCAGTCATAGAGTCGGA-3′
C′UL4A	NM_001008895.4	Forward	5′-CAGGCACAGATCCTTCCGTT-3′
		Reverse	5′-TGGTTTCTGTGTGCTGTGGT-3′
DDB1	NM_001923.5	Forward	5′-CAGTGTTTCGGGGTCCTCTC-3′
		Reverse	5′-AAGTCGCCCTTGGTCTTCAG-3′
RBX1	NM_014248.4	Forward	5′-ACTTCCACTGCATCTCTCGC-3′
		Reverse	5′-AAGTGATGCGCTCAGAGGAC-3′
HO-1	NM_002133	Forward	5′-CCAGCCATGCAGCACTATGT-3′
		Reverse	5′-AGCCCTACAGCAACTGTCGC-3′
NQO1	NM_000903	Forward	5′-TGATCGTACTGGCTCACTCA-3′
		Reverse	5′-GTCAGTTGAGGTTCTAAGAC-3′
Sp1	NM_138473.3	Forward	5′-ACAGTTCCAGACCGTTGATG-3′
		Reverse	5′-TGGTAGTAAAGTTCATAATT-3′
Nrf2	NM_001145412	Forward	5′-GCCATTCACTCTCTGAACTT-3′
		Reverse	5′-GGTGACAAGGGTTGTACCAT-3′
β-actin	NM_001101	Forward	5′-CAAGAGATGGCCACGGCTGCT-3′
		Reverse	5′-TCCTTCTGCATCCTGTCGGCA-3′

### Bioinformatics analysis of the promoter region

The sequence of the human *CUL4A* promoter and human *CUL4A* mRNA (GenBank accession number NM_001008895.4) were obtained from the National Center for Biotechnology Information. The prediction of putative transcription factor-binding sites on the *CUL4A* promoter was performed with the JASPAR database (http://jaspar.genereg.net/) (57). The mammalian conservation of Sp1-binding sites based on multiple alignments of 100 vertebrate species was mapped onto the human *CUL4A* promoter build on the UCSC genome browser (https://genome.ucsc.edu/) (58).

### Statistical analysis

Data are shown as the mean ± standard deviation. The significance of differences was examined using the Student's *t* test when two means were compared and a one-way ANOVA followed by the Bonferroni *post hoc* test when multiple comparisons were performed. *p* < 0.05 was considered to be significant (∗*p* < 0.05, ∗∗*p* < 0.01, ∗∗∗*p* < 0.001).

## Data availability

All data for this publication are included in the manuscript.

## Conflict of interest

The authors declare that they have no conflicts of interest with the contents of this article.
